# Selective Lithium Recovery via Stepwise Transition Metal Crystallization in a Natural Deep Eutectic Solvent

**DOI:** 10.1002/advs.202514509

**Published:** 2025-09-12

**Authors:** Jingxiu Wang, Jodie Yuwono, Yan Wang, Yanqiu Lyu, Sailin Liu, Tony Hall, Rong Zeng, Jianfeng Mao, Zaiping Guo

**Affiliations:** ^1^ School of Chemical Engineering The University of Adelaide Adelaide SA 5005 Australia; ^2^ Mawson Analytical Spectrometry Services Faculty of Sciences Engineering and Technology The University of Adelaide Adelaide SA 5005 Australia; ^3^ Department of Materials Science & Engineering City University of Hong Kong Kowloon Hong Kong 999077 China

**Keywords:** deep eutectic solvent, lithium‐ion batteries, scalability, selective lithium recovery, spent cathode recycling, universality

## Abstract

The global shift toward electric vehicles is driving unprecedented demand for lithium‐ion batteries, underscoring the urgent need for sustainable lithium (Li) recycling technologies. Deep eutectic solvents (DESs) have emerged as promising green alternatives to conventional leaching agents, yet challenges persist in preferential Li selectivity and process scalability. Here, a natural DES composed of choline chloride, lactic acid, and ascorbic acid (1ChCl‐10LA‐VC) is designed to overcome these limitations. In this system, LA provides high acidity, VC serves as a mild reductant, and ChCl promotes selective complexation and controlled precipitation of transition metals (TMs: Co, Ni, Mn, Fe). This synergistic formulation enables a stepwise separation mechanism: Li⁺ remains solubilized, while TMs undergo rapid reduction, complexation, hydrolysis, and eventual crystallization within one hour. The method is broadly effective across various cathode chemistries — including LiCoO_2_, LiNiO_2_, LiMn_2_O_4_, LiFePO_4_, and NCMs (NCM 111, NCM 523, NCM811) — and demonstrates exceptional selectivity, particularly for high‐Ni cathodes. Co‐dissolved Mn impurities are effectively removed via antisolvent crystallization, enabling high‐purity Li_2_CO_3_ recovery. When applied to black mass, the method achieves >96% Li leaching, >94% overall recovery, and excellent DES recyclability over four cycles. Furthermore, scale‐up to gram‐scale in a 2‐liter glass reactor confirmed process robustness and industrial feasibility.

## Graphical abstract

A biodegradable, natural deep eutectic solvent (DES) is designed for preferential lithium (Li) leaching from various cathodes under mild conditions (60 °C, 8 h). Compared to traditional acids and DESs, it achieves >96% Li leaching from black mass with minimal transition metal dissolution via a stepwise reduction, complexation, hydrolysis, and crystallization mechanism.

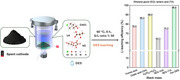

## 1 Introduction

The surging global demand for lithium (Li), driven by its essential role in lithium‐ion batteries (LIBs) used in electric vehicles and renewable energy storage,^[^ ^1^ ^]^ is creating a critical supply challenge.^[^ ^2^, ^3^ ^]^ Global Li demand is projected to increase by 18‐ to 20‐fold by 2050, reaching 983000 tons. Currently, Li is primarily extracted from energy‐intensive and geographically constrained sources such as hard‐rock ores and salt‐lake brines.^[^ ^4^ ^]^ Meanwhile, the rapid accumulation of waste LIBs has raised concerns about resource scarcity, environmental impact, and the need for a circular economy.^[^ ^4^ ^]^ By 2036, it is estimated that over 136 000 tonnes of waste LIBs will be generated annually, yet currently less than 10% are being recycled.^[^ ^5^ ^]^ Alarmingly, the global Li recycling rate remains below 1%,^[^ ^6^ ^]^ despite spent LIBs containing higher Li concentrations than natural ores. By 2030, Li demand is projected to exceed production by 10%,^[^ ^7^ ^]^ underscoring the urgent need for efficient, sustainable, and Li‐selective recovery technologies for recycling spent LIBs.

Hydrometallurgy is currently preferred over pyrometallurgy and direct recycling for recycling LIBs due to its energy efficiency and operational flexibility.^[^ ^8^, ^9^ ^]^ Unlike pyrometallurgy, which operates at high temperature leading to significant greenhouse gases and toxic emissions, hydrometallurgy uses chemical leaching but produces large volumes of wastewater that require careful treatment and disposal. Existing hydrometallurgical methods primarily target the recovery of valuable transition metals such as cobalt (Co) and nickel (Ni), resulting in significant Li loss – reported over 20% during co‐extraction.^[^ ^10^ ^]^ This not only undermines resource efficiency but also necessitates additional costly purification steps.^[^ ^11^ ^]^ Given the critical importance of Li, developing a process that enables preferentially selective Li recovery from spent LIBs is essential to enhance resource utilization and reduce reliance on primary raw materials.

Traditional Li‐selective leaching methods – using formic acid (FA),^[^ ^12^ ^]^ oxalic acid (OA),^[^ ^13^ ^]^ and phosphoric acid (PA) with H_2_O_2_^[^ ^14^ ^]^ – suffer from low efficiency, limited selectivity, poor Li product purity, safety concerns, and a lack of validation on real black mass (BM). Recently, deep eutectic solvents (DESs) have emerged as promising green leaching solvents due to their biodegradability, low toxicity, and tunability. In particular, several DES systems have demonstrated preferential Li leaching with minimal co‐dissolution of transition metal. For example, Tang et al. (2022) employed a DES with 5:1 molar ratio of ethylene glycol (EG) and oxalic acid dihydrate (OAD) (5EG‐1OAD) to leach LiNi_x_Co_y_Mn_1‐x‐y_O_2_ (NCM) at 90 °C, achieving 94.1% Li dissolution.^[^ ^15^ ^]^ However, oxalate‐based DES systems often suffer from compositional instability of OA. Yang et al. (2023) achieved Li separation from LiCoO_2_ (LCO) and NCM cathodes using a DES consisting of EG and tartaric acid (TA) under hydrothermal leaching at 120 °C. However, the leaching mechanism was inferred solely from LCO experiments, with cobalt tartrate assumed to be the precipitate, lacking direct experimental evidence.^[^ ^16^ ^]^

Despite these advancements, existing DES‐based studies also face challenges including limited tests on actual BM,^[^ ^17^, ^18^ ^]^ poor Li selectivity,^[^ ^19^ ^]^ harsh operating conditions,^[^ ^20^ ^]^ and low product purity,^[^ ^21^ ^]^ all of which hinder their industrial applicability.^[^ ^22^, ^23^ ^]^ Furthermore, most previous studies do not clarify why transition metals initially dissolve and later precipitate, or they provide only limited explanations based on simplified systems such as LCO. Although transition metal (TM or Me) precipitates are often calcined to obtain oxides, the solution‐phase transformation pathways remain poorly understood. This lack of mechanistic insight into DES‐metal interactions hinders further process optimization and scale‐up. Bridging this gap is essential for advancing DES‐based LIB recycling toward industrial application.

**Figure 1:**
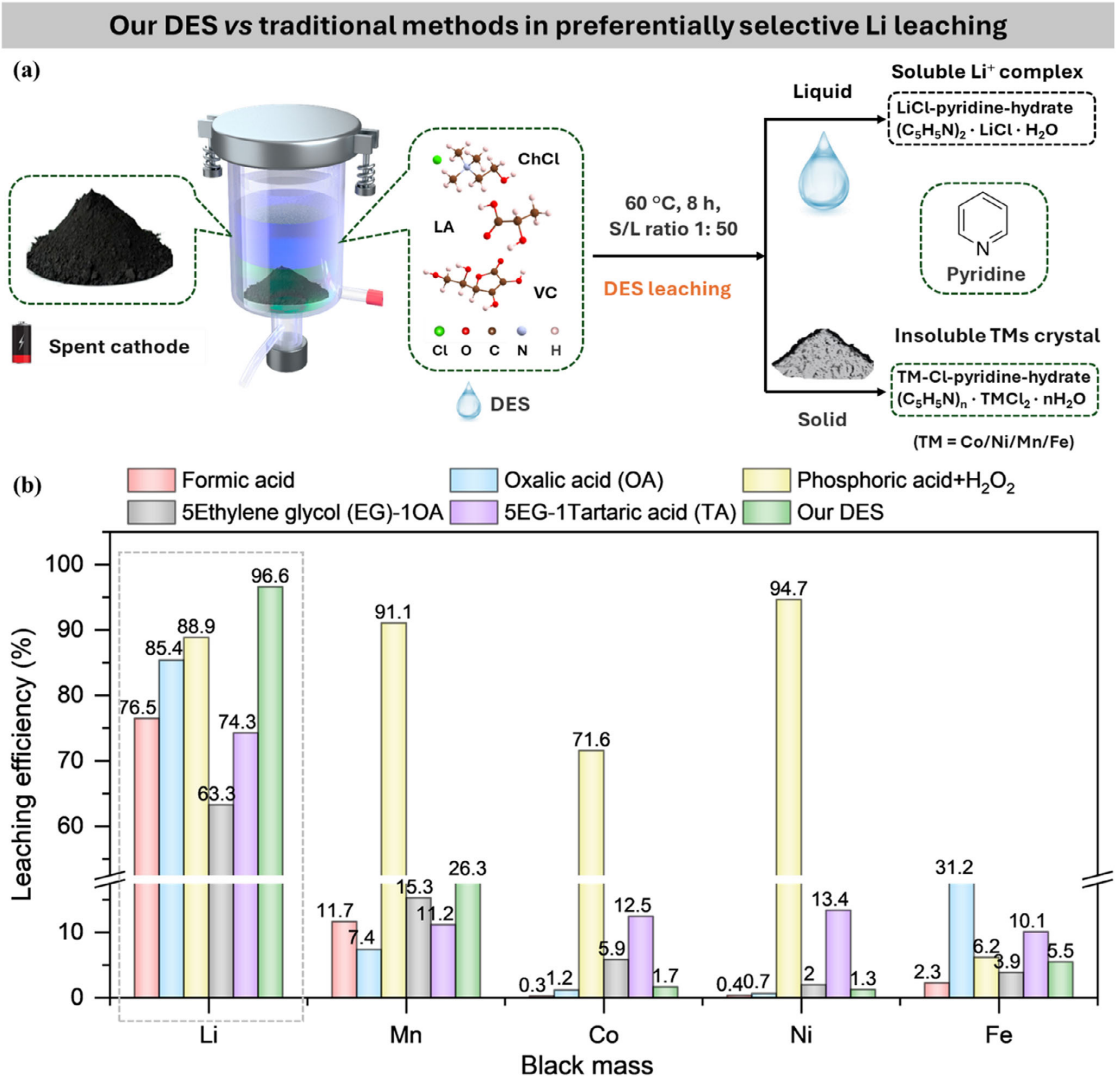
Comparison of our DES technology with conventional acid and DES methods in preferentially selective leaching of Li from spent cathodes. a) Schematic illustration of our DES technology for selective Li separation from transition metal(s). b) Our DES demonstrates superior Li leaching efficiency 96.6% from black mass over traditional acids^[^ ^12^, ^13^, ^14^ ^]^ and DESs.^[^ ^15^, ^16^ ^]^

In this study, we present a universal, scalable, and mechanism‐guided process for the selective recovery of Li from diverse spent LIBs cathodes using a DES composed of choline chloride, lactic acid, and *L*‐ascorbic acid (1ChCl‐10LA‐VC). Lactic acid and ascorbic acid are both naturally occurring, non‐toxic, and biodegradable agents with wide industrial applications.^[^ ^24^, ^25^ ^]^ This DES is effective across a broad range of cathode chemistries — including LCO, LiNiO_2_ (LNO), LiMn_2_O_4_ (LMO), LiFePO_4_ (LFP), and NCMs (111, 523, 811). Operated under mild conditions (60 °C, 8 hours, solid to liquid (S/L) ratio of 1: 50 g mL^−1^; **Figure** **1**a), this DES system achieves a high Li leaching efficiency (>96%) from black mass, while minimizing transition metals (Co, Ni, Fe <6%, and Mn = 26%) dissolution. Co‐dissolved Mn impurities are further removed by antisolvent crystallization, enabling the recovery of high‐purity Li_2_CO_3_ (93.5%). Based on our benchmark tests using black mass, the Li leaching efficiency achieved with our DES exceeds that of conventional leaching agents such as FA,^[^ ^12^ ^]^ OA,^[^ ^13^ ^]^ and PA with H_2_O_2_,^[^ ^14^ ^]^ as well as other DES systems (5EG‐1OAD,^[^ ^15^ ^]^ 5EG‐1TA^[^ ^16^ ^]^) (Figure 1b). Mechanistic insights, supported by density functional theory (DFT) and validated by UV‐visible (UV–vis) spectroscopy, X‐ray photoelectron spectroscopy (XPS), X‐ray diffraction (XRD), fourier transform infrared (FTIR) spectra, and gas chromatography‐mass spectrometry (GC‐MS) analyses, reveal a stepwise reduction–complexation–hydrolysis–crystallization pathway in which transition metals selectively precipitate while Li⁺ remains in solution. The DES system retains its selectivity and performance over at least four reuse cycles. The process has also been successfully scaled to the gram level and evaluated through techno‐economic analyses, demonstrating its feasibility and cost‐effectiveness for industrial implementation. Overall, this work provides a green, selective, and scalable solution for Li recovery and offers mechanistic insights critical for advancing DES‐based LIB recycling technologies.

## 2 Results and Discussion

### 2.1 Design of a DES for Preferentially Selective Li Leaching

A natural deep eutectic solvent (DES) was formulated using ChCl and LA in a 1:10 molar ratio, with 1% (w/v) VC added as a reducing agent, yielding the DES as 1ChCl‐10LA‐VC. The successful formation of this DES is demonstrated in Figure S1 (Supporting Information).

To evaluate the preferential Li leaching capability, LCO was used as a representative cathode material (properties provided in Figure S2, Supporting Information). The optimal conditions were determined at 60 °C, a solid to liquid (S/L) ratio of 20 g/L, and a duration of 8 hours (Figure S3, Supporting Information). Upon leaching, the black suspension in the glass bottle transformed into a light pink suspension, with distinct stratification observed after subsequent centrifugation (Figure S4, Supporting Information). Inductively coupled plasma mass spectrometry (ICP‐MS) analysis confirmed that lithium was enriched in the upper reddish liquid, while cobalt predominantly concentrated in the lower precipitate, achieving an in situ one‐step separation of lithium and cobalt. Li was nearly completely leached into the solvent, with a leaching efficiency of 98.2%, whereas Co dissolution was limited to 1.4%, resulting in a high Li selectivity of 98.6%.

When the leaching system was scaled up to a 50 mL volume, the leaching efficiencies of Li and Co remained similar at 96.4% and 1.51%, respectively. Further scaling up to 500 mL yielded comparable results, with Li and Co leaching efficiencies of 93.3% and 1.63%. The consistent leaching results across different scales (Figures S5 and S6, Supporting Information) underscore the scalability and potential feasibility of the process for future industrial applications.

**Figure 2:**
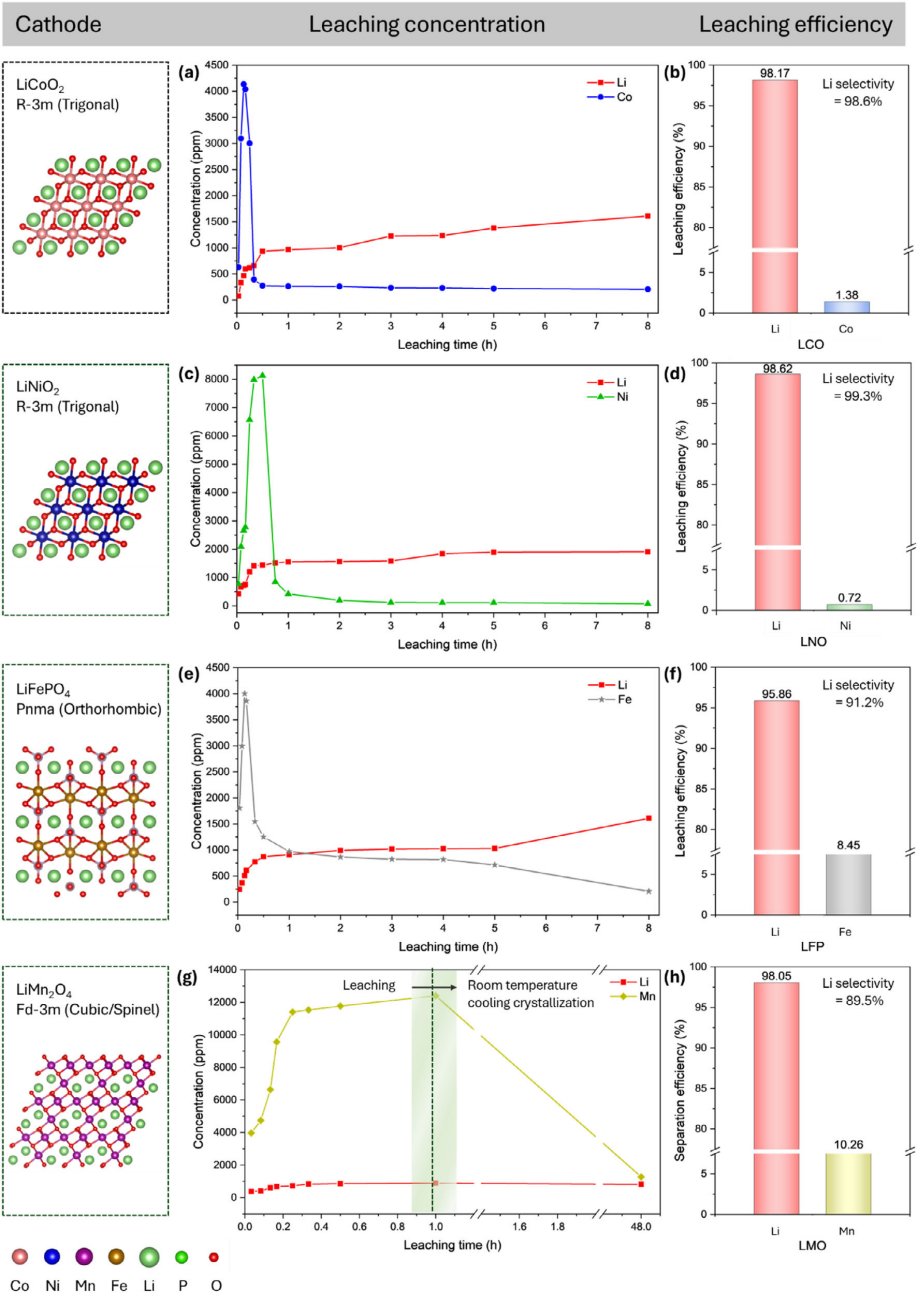
The leaching behaviors of commercial cathodes using the optimized DES process. a,b) LCO: (a) LCO leaching concentrations with time. (b) Leaching efficiencies of Li and Co. c,d) LNO: (c) LNO leaching concentrations with time. (d) Leaching efficiencies of Li and Ni. e,f) LFP: (e) LFP leaching concentrations with time. (f) Leaching efficiencies of Li and Fe. g,h) LMO: (g) LMO concentrations with time, combining leaching and room temperature crystallization. (h) Separation efficiencies of Li and Mn.

The leaching behaviors of commercial cathodes containing Li and individual transition metals—LCO, LNO, LFP, and LMO (Table S1, Supporting Information)—were also examined under the optimized leaching conditions outlined above. **Figure** **2** presents the metal leaching concentrations and leaching efficiencies after 8 hours for each of the cathode materials. Notably, Li concentration exhibited a continuous increase throughout the leaching process, while the concentrations of transition metal (Co/Ni/Fe/Mn) initially increased but then rapidly declined, particularly within the first 30 mins of leaching (Figure 2a,c,e,g). This behavior resulted in high Li leaching efficiency alongside limited transition metal dissolution (Figure 2b,d,f,h). Ultimately, a one‐step separation of Li from Co, Ni, Fe, and Mn was successfully achieved, demonstrating excellent Li selectivity. During leaching, distinct color changes in the suspensions were observed, indicating metal dissolution. For LNO, the suspension color changed from black to brown, with metal dissolution occurring within 1 hour (Figure S7, Supporting Information). In the case of LFP, the leaching solution remains black (Figure S8, Supporting Information), and Fe concentration continued to decrease over 8 hours, resulting in a low Fe leaching efficiency of 8.5%. For LMO, both Li and Mn dissolved completely within 1 h, yielding a clear solution (Figure S9, Supporting Information). A significant drop in Mn concentration was subsequently observed during cooling at room temperature, enabling a greater Li separation (Figure 2g,h). This was attributed to the formation of a high‐purity Mn precipitate (99.6%) with a needle‐like morphology exceeding 50 µm in length (Figure S10, Supporting Information). Overall, the selectivity of Li over transition metals follows the order: Ni > Co > Fe > Mn, highlighting the potential for preferential Li recovery.

### 2.2 Selective Li Leaching Mechanism

**Figure 3:**
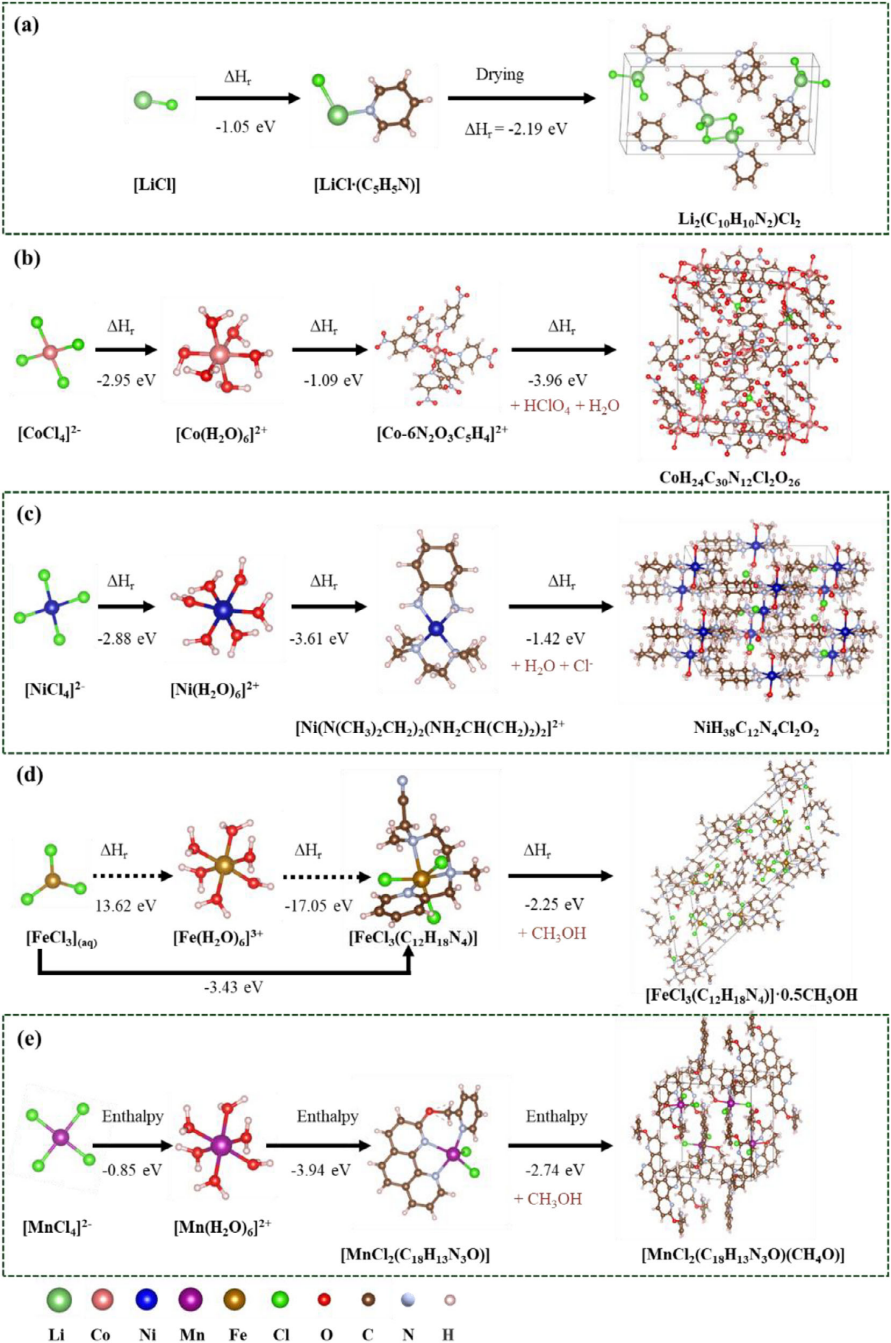
DFT‐calculated mechanistic pathways for selective lithium separation through transition metal crystallization using the designed DES. a) Li‐complex, b) Co‐crystal, c) Ni‐crystal, d) Fe‐crystal, and e) Mn‐crystal.

The leaching of the aforementioned cathodes using the designed DES is a complex process involving both solute transport and chemical reactions. Previous studies have confirmed the presence of hydrogen ions (H^+^) in acidic DES formulations.^[^ ^26^, ^27^ ^]^ In our DES system, both LA and VC can ionize to release H^+^, resulting in high acidity with a measured pH of ‐0.22. Compared to Li^+^, H^+^ has a smaller ion radius (38 pm vs 76 pm) and does not form direct bonds with oxygen atoms, making it more effective at disrupting Li‐O bonds than the stronger transition metal‐oxygen bonds. The reducing capability of VC plays a critical role in the system by reducing high‐valence transition metal ions (Me^3+^ or Me^4+^) to their soluble divalent forms (Me^2+^).^[^ ^28^ ^]^ Simultaneously, chloride ions (Cl^−^) from DES act as strong coordinating ligands, facilitating the formation of soluble transition metal complexes, which then selectively crystallize. This proposed mechanism is supported by comprehensive spectroscopic analyses, including UV–vis, XRD, XPS, and GC‐MS. **Figure** **3** presents the DFT calculated mechanistic pathways for Li complexation and transition metal crystallization.

#### 2.2.1 Lithium Reaction Pathway

Traditional lithium precipitation methods – such as solid Na_2_CO_3_, saturated Na_2_CO_3_ solution, or PO_4_^3−^ solutions‐ proved ineffective for precipitating the lithium filtrate, suggesting Li^+^ exists as a complex within the DES.^[^ ^29^, ^30^ ^]^ XRD analysis of the dried lithium filtrate (Figure S11a, Supporting Information) identified [LiCl∙(C_5_H_5_N)] as a key Li‐containing intermediate, a finding further supported by DFT calculations demonstrating Li^+^ complexation via LiCl (Figure 3a). FTIR spectra comparing the dried filtrate with the original solution (Figure S11b, Supporting Information) revealed significant spectral change: the emergence of a –C = N bond at (1578 cm^−1^, disappearance of –C = O (1752 cm^−1^) and –C–O (1211 cm^−1^) bonds, and notable shifts in –C–C, –C–N, and –C–H vibrations.^[^ ^31^ ^]^ In contrast, the FTIR spectra of all leachates closely resembled that of the original DES (Figure S12, Supporting Information), indicating that these changes occurred specifically during the drying process and were associated with the formation of pyridine‐coordinated LiCl complexes. GC‐MS analysis further detected pyridine derivatives in the leachate (Figure S13, Supporting Information), providing additional evidence of lithium complexation. It is well established that pyridine derivatives form strong coordination complexes with transition metals Co, Ni, Mn, and Fe, but only weakly interact with Li.^[^ ^32^ ^]^ This selective coordination offers a rational explanation for lithium remaining in solution, while transition metals undergo selective precipitation.

#### 2.2.2 Transition Metal Reaction Pathways

For transition metals, the selective precipitation mechanism involves stepwise complexation and ligand exchange processes. Initially, metal ions form chloride complexes of the type [Me(Cl)_x_]^n−^ (x = 3,4, n = 0,2), which subsequently transition into octahedral [Me(H_2_O)_6_]^2^ ^/3^⁺ species prior to crystallization. During LCO leaching, the filtrate exhibited a distinct colour change over time – from navy blue at 2 minutes, to dark purple at 5 minutes, light purple at 30 minutes, and finally reddish after 1 h (Figure S14a, Supporting Information). UV–vis analysis within the first 8 minutes confirmed the formation of tetrahedral [CoCl_4_]^2–^ species, characterized by absorbance peaks at 628, 665, and 696 nm (Figure S14b, Supporting Information). The emergence of a peak at 528 nm, attributed to octahedral [Co(H_2_O)_6_]^2+^,^[^ ^33^, ^34^ ^]^ indicates a phase transition from [CoCl_4_]^2–^ to [Co(H_2_O)_6_]^2+^.^[^ ^17^, ^35^ ^]^ After 10 mins, the disappearance of these characteristic peaks (Figure S14c, Supporting Information) corresponds to the onset of Co crystallization. XPS analysis of the resulting Co crystal confirms the reduction from Co^3+^ to Co^2+^, as evidenced by the Co 2p3/2 and 2p1/2 peaks along with satellite features characteristic of Co^2+^ (Figure S14d, Supporting Information), and ligand and adsorbed oxygen O 1s peaks (Figure S14e, Supporting Information). These results support the stepwise crystallization mechanism of Co‐precipitate, as depicted in Figure 3b. A similar trend was observed for LNO leachates, with the solution colour changing from light green (2 min) to dark green (30 min), and finally to reddish (Figure S15a, Supporting Information). This coincided with the rapid dissolution of Ni within the first 30 min, followed by a sharp decline in Ni concentration (Figure 2c). UV–vis spectra revealed the presence of [NiCl_4_]^2–^ with a characteristic peak at 400 nm,^[^ ^36^ ^]^ which disappeared after 45 mins as Ni crystallized (Figure S15b,c, Supporting Information), consistent with the pathway illustrated in Figure 3c. For LFP and LMO leachates, no UV–vis signals were detected (Figure S16, Supporting Information).^[^ ^37^ ^]^ However, similar leaching behaviors – including visible color changes, metal concentration profiles, and high Li selectivity – suggest analogous precipitation mechanisms. Specifically, species such as [FeCl_3_](aq) and [MnCl_4_]^2–^ are likely present and transition to stable crystalline phases (Figure 3d,e). Notably, Fe^2+^ was not detected in the LFP leachates, as it readily oxidizes to Fe^3^⁺ in solution – a finding confirmed by KMnO_4_ titration. This may explain the incomplete precipitation of Fe, attributed to the greater stability of Fe(II) pyridine complexes relative to those of Fe(III).^[^ ^38^ ^]^ Furthermore, XRD analysis (Figure S17, Supporting Information) confirmed the phase composition of the precipitated metal crystals, and FTIR spectra (Figure S18, Supporting Information) revealed the emergence of a C = N stretching band at 1578 cm^−1^.^[^ ^39^ ^]^ Collectively, these findings demonstrate that the DES components synergistically facilitate the stepwise reduction, complexation, hydrolysis, and crystallization of transition metals — thereby enabling the selective separation of Li from transition metals.

#### 2.2.3 Mechanistic Insights and Synergistic Effects

To further validate the synergistic effects of DES components, we conducted a series of experiments using stoichiometric amounts of individual chemicals and their combinations. These results revealed that no single component could achieve complete dissolution of LCO within 1 h (Figure S19, Supporting Information). However, the addition of VC to LA significantly enhance both Li and Co dissolution by effectively balancing acidity and reduction potential. While the binary mixture of 1ChCl‐10LA possesses moderate acidity and complexation ability, it lacks the necessary acidity and reducing power to achieve complete Li‐Co separation. The introduction of VC compensates for those deficiencies, enabling selective Li leaching while suppressing Co dissolution. ChCl plays a critical role in regulating Co precipitation by promoting controlled complexation and subsequent crystallization. The overall mechanism can be summarized as follows: Li^+^ remains in solution due to its preferential dissolution by abundant H⁺ ions. Concurrently, VC reduces high valence transition metal ions (Me^n+^) to their divalent forms (Me^2+^) during leaching. These Me^2+^ ions initially form unstable chloride complexes [MeCl_x_]^n−^ (x = 3,4; n = 0, 2), which quickly transform into more stable octahedral aquo complexes [Me(H_2_O)_6_]^2/3+^ in the presence of water.^[^ ^40^ ^]^ This stepwise transformation ultimately results in the crystallization of transition metal precipitates, enabling highly selective separation of Li from transition metals.

### 2.3 Extended Applicability on Ternary Cathodes

**Figure 4:**
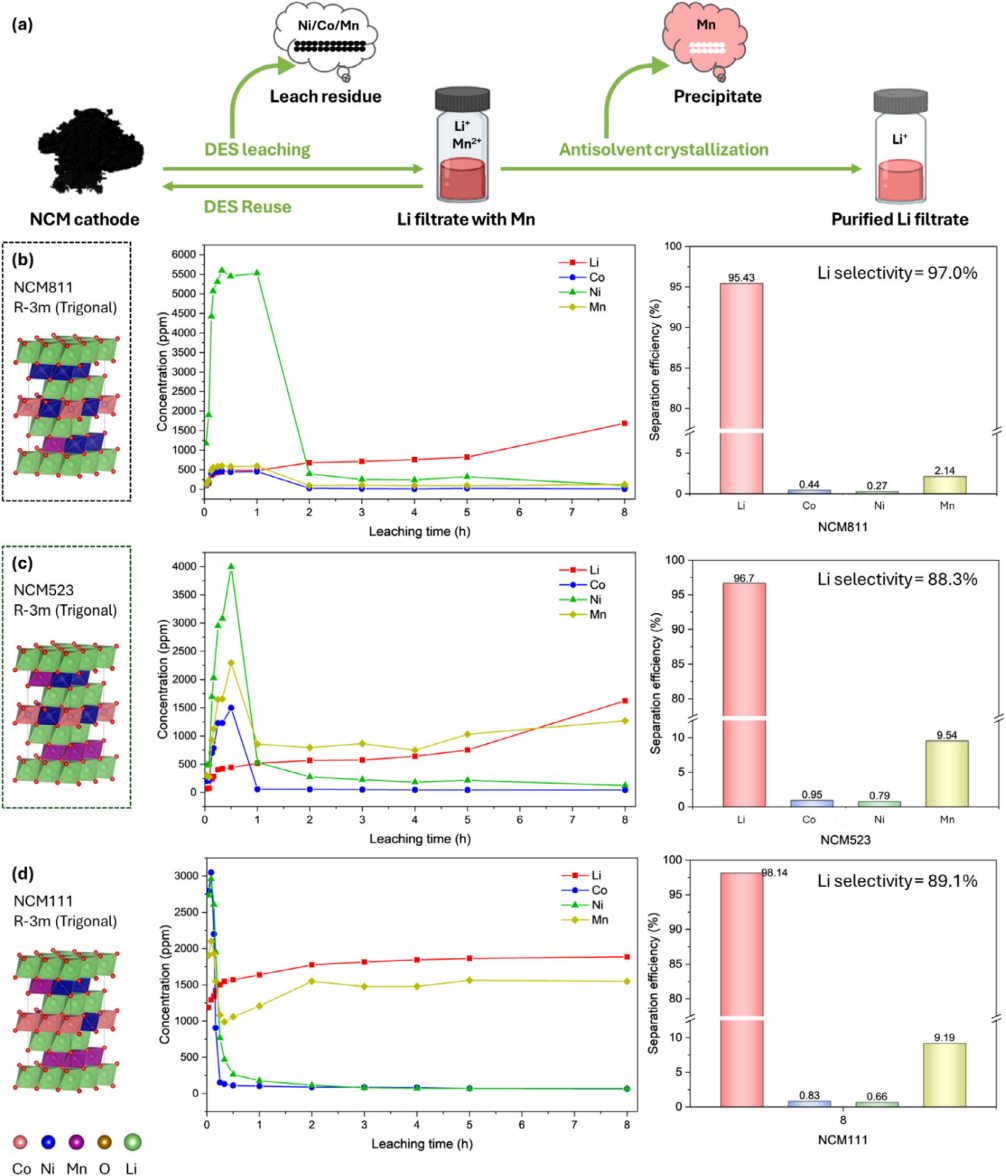
Universality of the designed DES for selective Li separation from ternary NCM cathodes. a) Schematic diagram illustrating the selective Li leaching process from ternary cathodes, integrating DES leaching, antisolvent crystallization, and purified Li filtrate for Li_2_CO_3_ recovery. b–d) Structural models of NCM cathode materials, metal concentration profiles during leaching, and separation efficiency after combining DES leaching and antisolvent crystallization. (b) NCM811; (c) NCM523; (d) NCM111.

Building on the selective Li leaching mechanism observed with individual transition metals, the designed DES was applied to ternary LIBs cathodes‐including NCM111, NCM523, and NCM811‐under optimized conditions to evaluate its broader applicability (**Figure** **4**). However, a notable challenge was encountered in high‐Mn cathode materials—significant co‐dissolution of Mn alongside Li, necessitating further purification steps to improve Li selectivity. This behaviour is likely attributed to the higher solubility of Mn‐containing crystalline phases in the DES environment, resembling the recrystallization behaviour previously observed in LMO leachates during cooling to room temperature.

To address Mn contamination, we explored various antisolvent options for Mn removal from a leachate containing 1887 ppm of Li^+^ and 1549 ppm of Mn^2+^. Among the tested solutions, 0.5 m oxalic acid (OxA) freshly dissolved in pure ethanol (no water added) proved to be the most effective (Figure S20, Supporting Information).^[^ ^41^ ^]^ XRD analysis confirmed the resulting precipitate as manganese oxalate dihydrate (PDF#97‐015‐0587). Following antisolvent crystallization, Mn impurity was significantly reduced, yielding a highly purified Li‐containing solution suitable for subsequent Li_2_CO_3_ recovery (Figure 4a). As shown in Figure 4b–d, the Li concentration steadily increased over an 8‐hour leaching period, while the concentrations of Ni, Co, and Mn initially rose within the first 2 hours before rapidly declining. This trend reflects the formation and subsequent precipitation of insoluble transition metal crystals. These concentration profiles aligned with the time‐dependent leaching efficiencies (Figure S21, Supporting Information), demonstrating the high selectivity of the process for Li. The results suggest that Co, Ni, and Mn initially dissolve but subsequently crystallize out of the solution, leaving Li^+^ predominantly in the solution. Applying 0.5 m OxA in ethanol as an antisolvent enabled high Li separation efficiency (>95%), while Co and Ni were effectively suppressed (<1.0%), and Mn removal remained moderate (<10%). Despite Mn separation efficiencies of 9.5% for NCM523 and 9.2% for NCM111, final Mn concentrations were reduced to 292 and 355 ppm, respectively. Importantly, this selective Li recovery method was especially effective for cathode materials with higher Ni content, aligning with the growing demand for high‐Ni NCM materials in next‐generation LIBs. Overall, these findings demonstrate that the combined DES leaching and antisolvent crystallization strategy offers a versatile and efficient method for the selective separation of Li from transition metals in various LIB cathode chemistries.

### 2.4 Establishment of a Prioritized Li‐Selective Leaching Method on Black Mass

**Figure 5:**
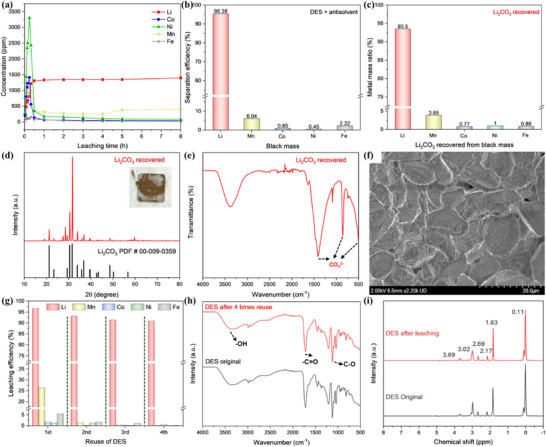
DES leaching of black mass followed by antisolvent crystallization for Li_2_CO_3_ recovery and solvent reuse. a) Metal concentrations during leaching. b) Separation efficiencies of metals combining DES leaching and antisolvent crystallization. c–f): Characterization of recovered Li_2_CO_3_. (c) Metal mass ratio. (d) XRD analysis. (e) FTIR spectrum. (f) SEM image. g–i): Reuse of DES leachate. (g) Metal leaching efficiencies over four times reuse. (h) FTIR of DES before and after 4 times of use. (i) ^1^H NMR of DES before and after 4 times of use.

To assess the selective Li leaching capability of the DES on black mass, a series of leaching experiments was conducted. During the leaching process, the Li concentration steadily increased (**Figure** **5**a), while the concentrations of transition metals initially rose and then declined, reaching equilibrium after approximately one hour. These trends correlated with observed changes in solution color and the time‐dependent leaching efficiencies of the metals (Figure S22, Supporting Information), confirming the high selectivity of the DES toward Li extraction. After 8 hours of leaching, the resulting leachate contained 399 ppm of Mn and less than 50 ppm of other TMs. To further remove the impurities, antisolvent crystallization was carried out by adding a 0.5 m OxA solution in pure ethanol (no water added) to the Li‐rich solution at a volume ratio of 3:1. After mixing at room temperature for 24 hours, a white precipitate was generated, achieving 85.6% Mn removal and 50.3%, 64.6%, and 59.5% removal of Co, Ni and Fe, respectively, with minimal Li loss of only 1.3% (Figure S23, Supporting Information). This combined DES leaching and antisolvent crystallization approach yielded a high overall Li separation efficiency of 95.4% (Figure 5b). Following ethanol recovery by distillation, the purified Li‐containing filtrate was calcined at 120 °C for 24 hours, then heated at 500 °C for 3 hours to obtain Li_2_CO_3_. The recovered Li_2_CO_3_ exhibited a high purity of 93.5%, as demonstrated by ICP analysis (Figure 5c). XRD analysis confirmed the phase identity and crystallinity, matching the standard card of Li_2_CO_3_ (JCPDS 00‐009‐0359) (Figure 5d). The FTIR spectrum showed characteristic carbonate absorbance peaks in the 1600–500 cm^−1^ range (Figure 5e),^[^ ^31^ ^]^ and the scanning electron microscope (SEM) image revealed a scaly morphology with particle size around 10 µm in size (Figure 5f). Overall, the complete lithium recovery from BM to Li_2_CO_3_ was calculated at 94.2%.

From both economic and sustainability perspectives, maximizing solvent reuse is essential. To evaluate the reusability of the designed DES, multiple leaching cycles were conducted. After four successive cycles with replenishment of VC, the DES consistently demonstrated excellent selective leaching performance. Li leaching efficiency remained above 90.9%, while the dissolution of transition metals was effectively suppressed to below 2% after the second cycle (Figure 5g). FTIR spectra of the DES before and after four reuse cycles revealed nearly identical characteristic peaks (Figure 5h), indicating minimal structural or chemical changes. The proton nuclear magnetic resonance (^1^H NMR) spectra also showed no obvious variations (Figure 5i), further confirming the preservation of the DES's chemical integrity throughout repeated use. Therefore, no degradation of DES components was detected.

Additionally, the leaching residue was calcined to produce a mixed oxide comprising Ni, Co, Mn, and Fe, with Ni and Co being the dominant components (Figure S24, Supporting Information). The calcined material exhibited a high purity of 99.7%, making it suitable for use in the preparation of commercial products. Future work will focus on the selective recovery of individual transition metal oxides from this mixed residue to support the production of new LIB cathode materials, further improving the economic viability and environmental sustainability of this process.

### 2.5 Practical Recycling Loop and Techno‐Economic‐Environmental Metrics Analysis

**Figure 6:**
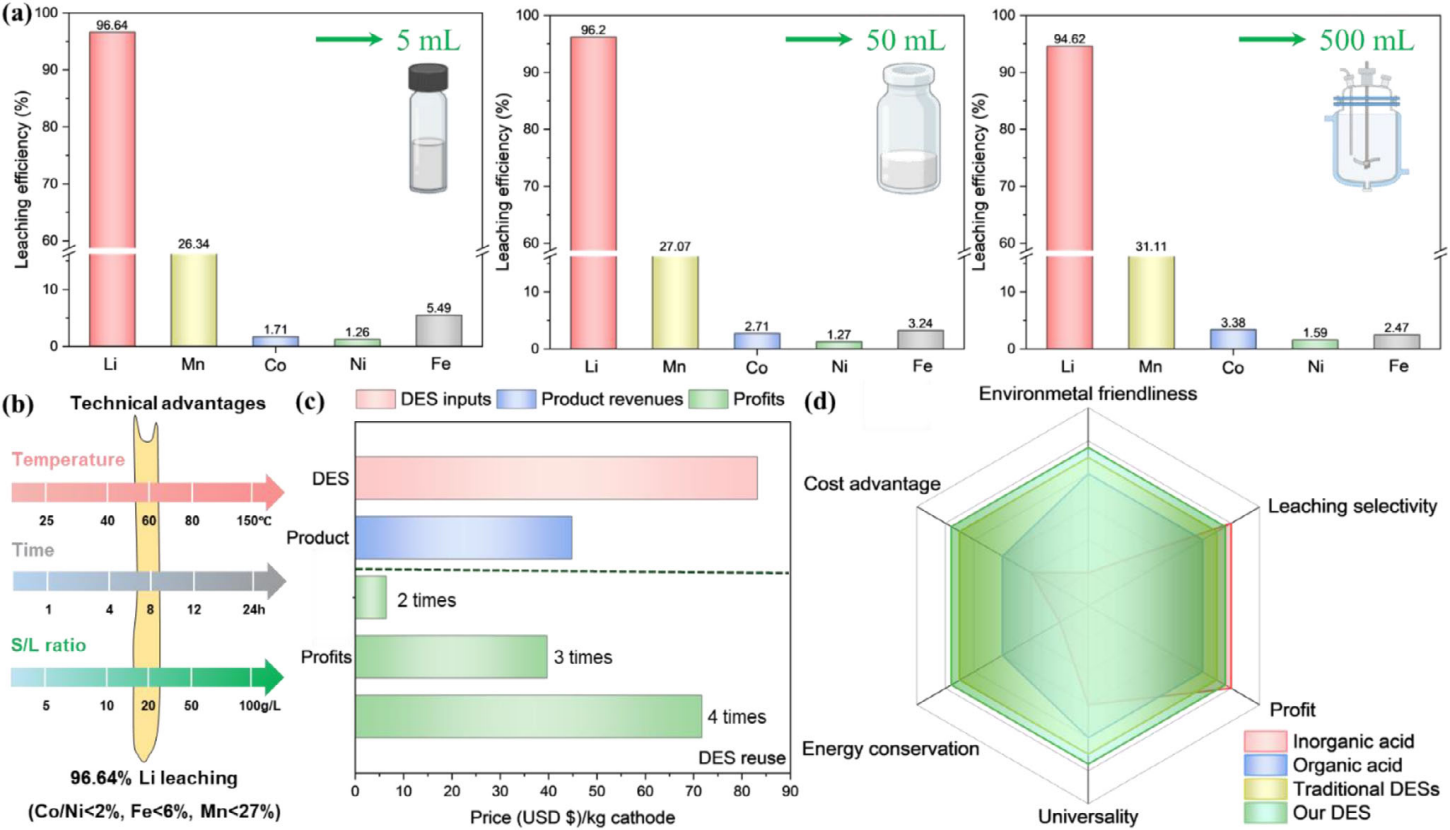
Scaling up the DES leaching process and assessment of its industrial applicability. a) DES leaching efficiencies from 5 to 50 mL and 500 mL. b) Technical advantages. c) Economic evaluation. d) Merits indication.

Since Tran et al. (2019)^[^ ^21^ ^]^ pioneered the use of DESs for extracting valuable metals from spent LIBs, research in this area has steadily progressed. However, a major challenge remains in translating DESs‐based metallurgical process to industrial‐scale application.^[^ ^42^ ^]^ To address this, we scaled up the BM leaching system from an initial 5 mL volume to a 50 mL, and subsequently to 500 mL using a 2‐litre glass reactor (Figure S25, Supporting Information). Prior to leaching, water was used to test and calibrate temperature and mixing settings. During operation, temperature was monitored with a thermometer, BM was added via a funnel after the DES reached the set temperature, and residual DES was used to rinse the funnel. A top‐mounted mixer ensured uniform mixing throughout the reaction. Across all scales, consistent Li leaching efficiencies were achieved – 96.64%, 96.20%, and 94.62%, respectively (**Figure** **6**a). These results demonstrate strong process scalability and indicate promising potential for industrial implementation. To further bridge the gap between laboratory research and practical deployment, we developed a closed‐loop circular recycling process suitable for large‐scale trials (Figure S26, Supporting Information). A comprehensive evaluation of this system was conducted from technical, economic and environmental perspectives to assess its overall feasibility and sustainability.

#### 2.5.1 Technical Advantage

Compared with other DESs that use similar hydrogen bond acceptor (HBA) or hydrogen bond donor (HBD), most reported systems do not prioritize Li selectivity. Typically, they result in co‐dissolution of transition metals, necessitating additional, often harsh, separation processes, and do not allow for DES reuse (Table S2, Supporting Information). To date, only four DES formulations have demonstrated preferential Li selectivity. While certain conventional acids – such as formic acid, oxalic acid, and phosphoric acid (when combined with H_2_O_2_) – have shown some selectivity for Li, our DES system (1ChCl‐10LA‐VC) significantly outperforms these alternatives under notably milder conditions, operating efficiently at 60 °C for 8 hours (Figure 6c). This DES exhibits exceptional Li leaching selectivity across various cathode chemistries, including high‐Ni NCM811, which is increasingly prevalent in next‐generation LIB. Key advantages for industrial application include its strong acidity (pH ‐0.22) and moderate density (1.1208 g mL^−1^ at room temperature), which collectively contribute to lower energy consumption and enhanced leaching efficiency.^[^ ^43^ ^]^ The process is streamlined, requiring only two main steps without the use of strong acids or alkalis, and enables simplified separation. These features present a technically viable and sustainable pathway for the recovery of critical metal sources from spent LIBs, supporting the future industrialization of closed‐loop battery recycling for EVs.

#### 2.5.2 Preliminary Economic Assessment

To assess the economic viability of the proposed selective Li recycling process, we conducted a cost‐benefit analysis based on the treatment of 1 kg of black mass to recover Li_2_CO_3_ and mixed TM oxides. Material costs typically represent the largest component of total recycling expenses; therefore, this analysis utilizes the lowest bulk reagent prices sourced from Alibaba (USD $ in ton‐scale quantities): choline chloride (ChCl, 99%) at 1$/kg, L‐lactic acid (LA, 85%) at 1.2$/kg, and L‐ascorbic acid (VC, 99%) at 2.5$/kg. Processing 1 kg of BM using 50 L DES requires approximately 7 kg of ChCl, 45 kg of LA, and 0.5 kg of VC, totaling 62.25$ in reagent cost. Based on mass balance calculations, this process yields 244.2 g Li_2_CO_3_ (valued at 60$/kg) and 500 g mixed TM oxides (valued at 50$/kg), generating a combined product value of 39.65$. This suggests that two cycles of DES reuse can lead to net profit. Upon extending reuse to four cycles—supplemented only by incremental VC addition at each cycle (totalling 3.75$)—a net profit of 83.2$ is realized (Figure 6d). Comparative cost analyses with other DESs in processing of 1 kg cathode (Table S3, Supporting Information) demonstrate the significant cost‐effectiveness of this DES compared to other DES‐based methods. While the recovered transition metals are currently obtained as mixed oxides suitable for direct sale or use as cathode precursors, future work will target the selective recovery of individual TM oxides to further enhance market value and industrial applicability.

#### 2.5.3 Environmental Implication

The increasing deployment of LIBs, particularly those using ternary NCM cathodes rich in nickel, cobalt, and manganese, presents severe environmental risks to water, soil, and human health. The selective Li recovery process developed in this study mitigates these concerns through the use of a DES system designed with environmental safety and sustainability in mind. Compared to other HBAs, ChCl‐based DESs exhibit lower toxicity than alternatives such as N,N‐diethylammonium chloride. Among HBDs, LA presents a safer profile relative to glycolic acid or citric acid.^[^ ^10^ ^]^ In addition, VC is a biodegradable agent. These three components‐ChCl, LA and VC – are all naturally derived and readily biodegradable, resulting in a significantly reduced environmental footprint compared to most DES formulations. Notably, lactic acid is produced via a green fermentation process, and Vitamin C is industrially manufactured through biotechnological processes, with an annual global production exceeding 130 000 tons. The system also supports DES regeneration:^[^ ^43^ ^]^ following metal precipitation, the DES can be effectively reused by replenishing VC, maintaining high lithium leaching performance across multiple cycles while suppressing further transition metal dissolution. In contrast to conventional inorganic acids‐based recycling methods, this DES system minimizes hazardous chemical use, reduces secondary pollution, and aligns with green chemistry principles and circular economy practices (Figure 6e).

The above analysis confirms that our DES‐based process offers a technically robust, economically viable, and environmentally sustainable method for the selective recovery of Li from spent LIB cathodes. By maintaining high selectivity and leaching efficiency under mild conditions, supporting solvent reuse, and utilizing biodegradable, low‐toxicity components, this method effectively bridges the gap between laboratory research and industrial‐scale implementation, advancing the development of green and circular battery recycling technologies.

## 3 Conclusion

This study presents a universal, scalable, and mechanism‐guided approach for the selective recovery of Li from spent LIB cathodes using a newly formulated deep eutectic solvent (1ChCl‐10LA‐VC). Operating under mild conditions (60 °C, 8 h), the system achieves a high Li leaching efficiency of 96.6% from black mass – significantly outperforming conventional acids and DES‐based methods. The process exhibits broad applicability across multiple cathode chemistries, including LCO, LNO, LFP, and LMO, while maintaining transition metal dissolution below 11%. Mechanistic investigations, supported by DFT calculations and validated by UV–vis, XPS, GC‐MS, and XRD analyses, elucidate a stepwise separation pathway in which Li^+^ remains in solution while transition metals (Co/Ni/Mn/Fe) undergo reduction, complexation, hydrolysis, and crystallization. Extension of this strategy to NCM cathodes demonstrated particularly high efficiency for high‐Ni compositions. The co‐dissolved Mn was effectively removed via antisolvent crystallization, enabling the production of high‐purity Li_2_CO_3_ (93.5%) from black mass. The DES showed excellent reusability for at least four cycles with minimal performance loss. Scale‐up experiments from 5 mL to 500 mL demonstrated consistent results, confirming the robustness and industrial scalability of the process. A closed‐loop recycling scheme was established, offering a well‐balanced solution in terms of technical feasibility, economic viability, and environmental sustainability. Overall, this work provides a feasible and sustainable solution for selective lithium recovery, addressing key challenges in battery recycling and contributing to the development of a circular economy for critical raw materials.

## Conflict of Interest

The authors declare no conflict of interest.

## Supporting information



Supporting Information

## Data Availability

The data that support the findings of this study are available from the corresponding author upon reasonable request.
