# Supplementary material for: Selective Lithium Recovery via Stepwise Transition Metal Crystallization in a Natural Deep Eutectic Solvent
Source: Adv Sci (Weinh). 2025 Sep 12;12(45):e14509. doi: 10.1002/advs.202514509 (PMC12677677; doi:10.1002/advs.202514509)
Supplement: Supplementary file 1 — Supporting Information [file ADVS-12-e14509-s001.docx]

**Supporting Information**

**Selective Lithium Recovery via Stepwise Transition Metal Crystallization in a Natural Deep Eutectic Solvent**

Jingxiu Wang^1^, Jodie Yuwono^1^, Yan Wang^1^, Yanqiu Lyu^1^, Sailin Liu^1^, Tony Hall^2^, Rong Zeng^1^,

Jianfeng Mao^1,^*, Zaiping Guo^1,3,^*

^1^School of Chemical Engineering, The University of Adelaide, Adelaide, SA 5005, Australia.

^2^Mawson Analytical Spectrometry Services, Faculty of Sciences, Engineering and Technology, The

University of Adelaide, Adelaide, SA, 5005, Australia.

^3^Department of Materials Science & Engineering, City University of Hong Kong, Kowloon, Hong Kong 999077, China

* Corresponding authors: [jianfeng.mao@adelaide.edu.au](mailto:jianfeng.mao@adelaide.edu.au), [zaipiguo@cityu.edu.hk](mailto:zaipiguo@cityu.edu.hk)

**Experimental Section**

**Materials and chemicals**

***Materials.*** The cathode materials of LCO, LNO, LFP, LMO, NMC111, NCM523, and NCM 811, were purchased from Sigma-Aldrich, and black mass was provided by Iondrive Technologies. These samples are used for the leaching experiments as received. At first, cathode materials were digested with aqua regia (concentrated HCl: HNO_3_ at 3:1 ratio) to determine the content of each metal (**Table S1**).

***Chemicals****.* Lactic acid (LA, ≥85%), choline chloride (ChCl, ≥98%), L-ascorbic acid (Vitamin C, ≥99%), formic acid ([≥98%](https://www.sigmaaldrich.com/AU/en/product/sigald/33015)), oxalic acid (≥99%), phosphoric acid (≥85%) and H_2_O_2_ (30%) were purchased from Sigma-Aldrich. All chemicals used in the experiment were of analytical grade and used without further purification.

**Preparation of deep eutectic solvents**

As reported previously, the DESs were prepared by mixing ChCl and LA at different molar ratios in a glass vial with a screw cap and heating up at 60 ^o^C until a transparent and homogeneous liquid mixture was attained. After that, the formed liquid was cooled down at low temperature. Prior to experiments, a certain amount of VC was added into the mixture and stirred at 60 ^o^C until complete dissolution to form a ternary DES.

**DESs leaching and separation of Li from spent cathodes**

***DESs leaching.*** The leaching experiments were conducted by adding an appropriate amount of cathode materials and 5 mL DES to a sealed glass vial and stirred for a certain time at a specific temperature at 1000 rpm. Different molar ratios of ChCl to LA (1:1-1:16), amount of VA (0-0.1 g), temperatures (40–90 ^o^C), leaching time (1–24 h), solid-liquid ratios (S/L ratio, 10-30 mg/mL) are tested. After leaching, the mixtures were centrifuged at a 10000 rpm for 3 min, with the liquid phase was filtrated by the 0.22 µm parafilm to obtain filtrate while the solid was washed with ethanol three times followed by drying at 90 ^o^C for 24 hours. The concentrations of the metallic ions in the filtrate and residue were determined using an inductively coupled plasma mass spectrometer (ICP-MS, Agilent 7900). The leaching efficiency (LE, wt%) of metal, the leaching selectivity (LS) of Li over transition metal(s), the separation efficiency (SE, %), the purity (Pu, wt%) of recovered metal product and recovery yield (RE, wt%) are calculated by the formula below:

| $LE(\%)=\frac{C_{Me, DES}\times V}{m_{Me,Original}}\times100$ | (1) |
| --- | --- |
| $LS(\%)=\frac{{LE}_{Li}-{LE}_{Co}-{LE}_{Ni}-{LE}_{Mn}-{LE}_{Fe}}{{LE}_{Li}}\times100$ | (2) |
| $SE(\%)=LE(\%)\times(1-precipitation\%)$ | (3) |
| $Pu(\%)=\frac{m_{Me}}{m_{Li}+m_{Co}+m_{Ni}+m_{Mn}+m_{Fe}}\times100$ | (4) |
| $RY(\%)=\frac{m_{Me}}{m_{Me, Added}}\times100$ | (5) |

where C*_Me,DES_*, V, and m*_Me,Original_* represent the concentration of metal Me in DES measured by ICP-MS, the volume of DES, and the mass of metal Me contained in the original added cathode material. Also, m*_Me_*, m*_Li_*, m*_Co_* and m*_Me,Original_* is the mass of metal Me, metal Li, metal Co, and metal Me added into the process, respectively. All data related to the leaching concentration, leaching efficiency, and separation efficiency were measured in triplicate, with an observed error margin within 4%.

***Antisolvent crystallization and DES reuse.*** A series of antisolvents with a set volume ratio were added to the leachate filtrate, followed by stirring at room temperature for 3 h. The resulting mixture was centrifuged at 10000 rpm for 3 mins, and the liquid and solid are collected separately for analysis. The solution after antisolvent crystallization was used for lithium recovery. The recovered product was dried in a vacuum oven at ~120 °C for 24 h, and then calcined at 500 °C for 3 h in a muffle furnace to obtain high-purity Li_2_CO_3_. To evaluate DES reusability, the leaching filtrate was reused for new leaching cycles. New cathode material was added to the reused DES under the same optimal leaching conditions, and the process was repeated for four cycles. The DES before and after each cycle was analysed for the compositional changes.

**Materials characterization**

***FT-IR spectroscopy****:* The FT-IR spectra of solid and liquid samples were recorded in the range of 400–4000 cm^−1^ at a resolution of 1 cm^−1^ using a Fourier transform infrared (FT-IR) spectrometer (Nicolet 6700, Thermo Fisher) using KBr discs.

***^1^H -NMR measurements****:* The chemical shifts of the DESs samples were detected by nuclear magnetic resonance hydrogen spectroscopies (^1^H NMR) spectrometer (Agilent 500 MHz), using deuterium oxide (D_2_O) as the external standard reagent.

***UV–vis spectra*** were collected with a resolution of 1 nm in the range of 200-900 nm by using a dual-beam Ultraviolet-visible Spectrophotometer (UV-vis, Shimadzu UV-2700). All measurements were conducted against the DES blank, and no dilution has been carried out.

***X-ray diffractometer (XRD)****:* The crystal structure of the cathodes, leached residues, and recovered products were characterised using an X-ray diffractometer (XRD, Rigaku MiniFlex 600, Japan) with Cu Kα radiation.

***Scanning electron microscopy (SEM):*** The micromorphology was observed by scanning electron microscope (JEOL JSM-7100F, Zeiss SIGMA FESEM, Germany), employing 18 kV accelerating voltage.

***Particle size analysis:*** The sample was measured using a laser particle size analyser (Mastersizer 2000, Malvern).

***Thermogravimetry and Differential Scanning Calorimetry (TG/DSC):*** Differential scanning calorimetry (DSC) and thermogravimetric methods (TG) (Mettler Toledo) were used to evaluate the thermal stability of DESs within a temperature range of 20−400 °C with a heating rate of 5 °C/min in nitrogen.

***X-ray photoelectron spectroscopy (XPS):*** The valance states of the metals in cathodes, leach residues and roasted products were obtained by an ESCALAB Xi+ (Thermofisher Scientific, USA) with an Al Kα X-ray source (hν =1486.6eV), the binding energies were calibrated by C 1s peak at 284.8 eV.

***Gas chromatography-mass spectrometry (GC-MS):*** GCMS was conducted on an Agilent 5973B/7890B instrument with a 30m Agilent HP-5MS capillary column. Helium carrier gas at 1mL/min was used with a 200:1 split and 1µL injection. The oven was programmed to hold at 50 ^o^C for 1 minute before ramping to 300 ^o^C at 10 ^o^C/min and being held for 14 minutes. Data was acquired over a scan range of 50:500 amu at approximately 3 scans per minute.

**Computational methods**

Density functional theory (DFT) calculations were performed using the Projector Augmented Wave (PAW) method^1,2^ as implemented in the Vienna Ab initio Simulation Package (VASP).^3,4^ The calculations were completed with a plane-wave cut-off energy of 500 eV and a single Gamma k-point. The electronic self-consistent calculation was converged to 1×10^-5^ eV and ionic relaxation steps were performed using the conjugate-gradient method (IBRION=2) and continued until the total force on each atom dropped below a tolerance of 1×10^-2^ eV/Å. The generalised gradient approximation (GGA) was used for the exchange correlation functionals as parameterized by Perdew-Burke-Ernzerhof (PBE).^5^ The dispersion correction was also included in this study by using DFT D-3 method.^6^ The identification of precipitates has been conducted using the screening of all possible crystal structures based on the XRD features. Based on the identified crystal structures, we then proposed possible reaction pathways for leaching and precipitation reaction. Additionally, intermediates in the leaching solution were verified using GC-MS investigations.


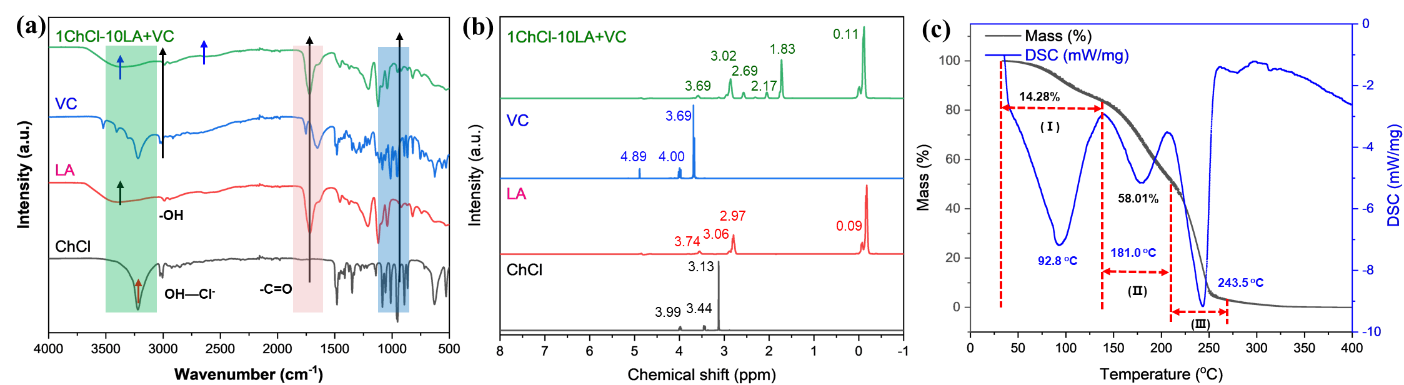


**Figure S1.** (a) FT-IR, (b) ^1^H NMR spectra, and (c) TG-DSC analysis of DES (1ChCl-10LA-VC).

**Figure S1a** shows the presence of characteristic peaks of the prepared DES in FT-IR spectroscopy, indicating the interactions of hydrogen bonds between 3500 and 2500 cm^-1^. Specifically, the shifting of the O–H stretching vibration from 3461 cm^-1^ in ChCl to 3501 cm^-1^, accompanied by broadening of absorption bands of VC in the range of 3206 cm^-1^ to 3526 cm^-1^ in DES (green region).^7^ The blueshift of the C=O vibration from 1740 cm^-1^ in LA to 1711 cm^-1^ in DES (pink region) is attributed to the strong electronegativity of Cl^-^ and the induction effect. Additionally, the blue region below 1200 cm^-1^ represents different kinds of C-H, C-O and CH_3_ vibrations. These changes confirm the formation of hydrogen bonds within the DES.

**Figure S1b** shows that the ^1^H NMR spectra of individual components (ChCl, LA and VC) align well with the literature results.^8^ However, the substantial shifts in peak positions after the DES formation indicate the strong interactions of hydrogen bonding. For example, the hydroxyl group peak in LA shifted from 2.97 ppm to 3.02 ppm in DES, further confirming hydrogen bonding in the DES structure.

In **Figure S1c**, TG/DSC revealed a three-stage decomposition process for DES. The first stage is water evaporation <130 ^o^C, and the mass loss at 14.28% was roughly the water mass in the system. The second stage is LA and VC decomposition at 150-210 ^o^C, with T_peak_ at 181.0 °C. Finally, ChCl was decomposed at 210-260 ^o^C, marking the final stage of thermal degradation. Therefore, it will be safe to conduct the leaching experiments below 100 ^o^C in a closed vessel to keep the water content constant.


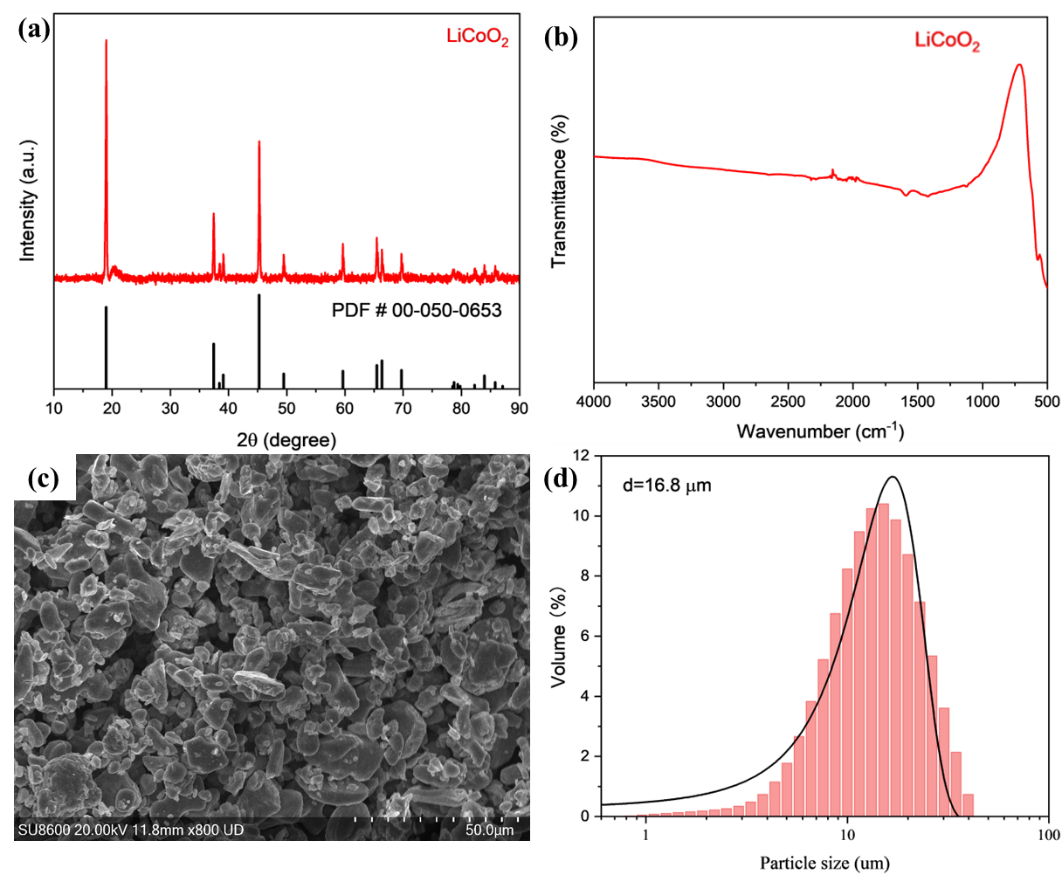


**Figure S2.** (a) XRD patterns, (b) FTIR spectra, (c) SEM images and (d) PSD analysis of LCO.

**
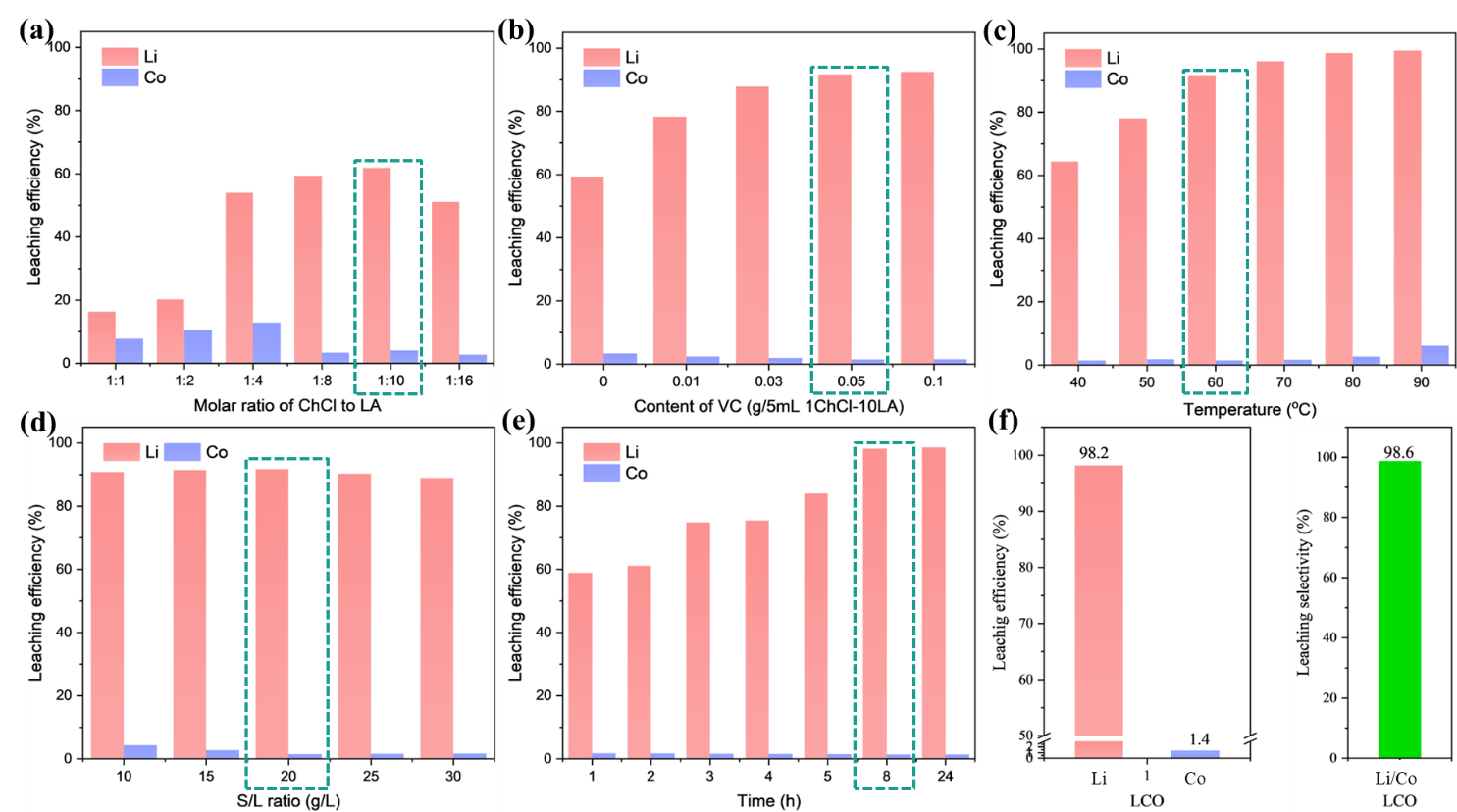
**

**(c)**

**Figure S3.** Effect of (a) Molar ratio (no VC, 60oC, 20 g/L, 8 h. ); (b) VC content (1:10 molar ratio of ChCl to LA, 60oC, 20 g/L, 8 h.); (c) Temperature (1:10 molar ratio of ChCl to LA, 0.05 g VC/ 5 mL 1ChCl-10LA, 20 g/L, 8 h.); (d) S/L ratio (1:10 molar ratio of ChCl to LA, 0.05 g VC/ 5 mL 1ChCl-10LA, 60oC, 8 h.); and (e) time (1:10 molar ratio of ChCl to LA, 0.05 g VC/ 5 mL 1ChCl-10LA, 60oC, 20 g/L.) on the leaching of LCO; and (f) leaching results under optimized conditions.

The optimization of LCO leaching results show that the leaching efficiency of Li and Co initially increases and then decreases with a higher molar ratio of ChCl to LA, reaching an optimal ratio at 1:10 (**Figure S3** a). To further increase Li selectivity and leaching efficiency, different amounts of VC were added to 5 mL solution, with 0.05g VC proving to be the most effective (**Figure S3** b). As the temperature increased, the leaching efficiency of both Li and Co gradually increased, with 60 ^o^C being selected as optimal for balancing leaching selectivity and industrial feasibility (**Figure S**3 c). The influence of varying S/L ratios showed that Li extraction increased first and then decreased, while Co extraction exhibited a decreasing trend (**Figure S3** d). This gives a highest selectivity of Li over Co at a ratio of 20 g/L, which was chosen for subsequent studies. Leaching time significantly affected the DES leaching performance, with the leaching efficiency of Li continuously increasing until complete dissolution was achieved after 8 hours (**Figure S3** e). Herein, the optimized conditions are a 1:10 ChCl-LA, 0.05 g VC in 5 mL DES, 60 ^o^C and 20 g/L S/L ratio for 8 hours. Li was completely leached out at 98.2%, while Co dissolution was controlled at 1.4%, resulting in a high Li selectivity of 98.6% (**Figure S3** f).

**
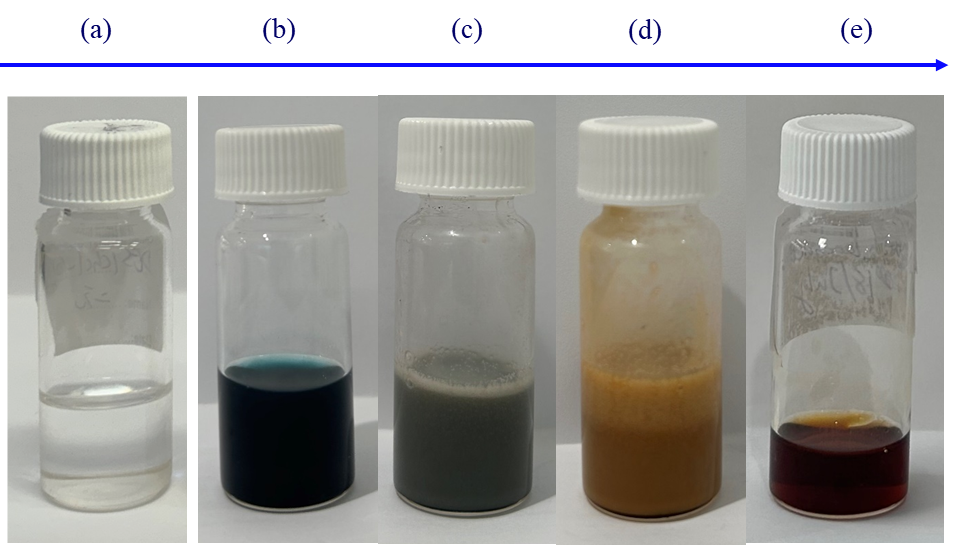
**

**Figure S4.** Photograph of 5 mL (a) DES; (b) DES with newly added LiCoO_2_. (c) the mixture after 30 mins; (d) the mixture after optimal leaching. (e) the supernatant after centrifugation and filtration.

**
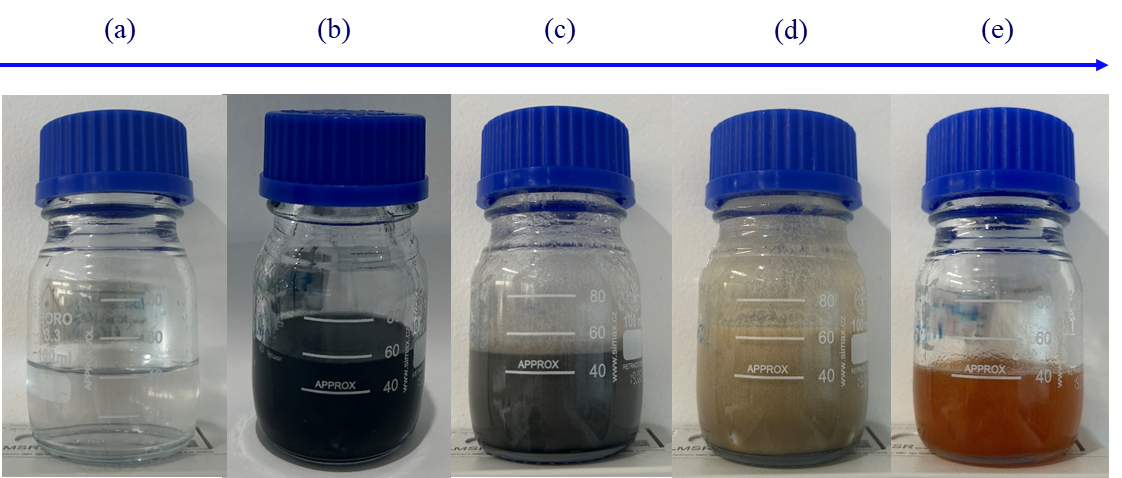
**

**Figure S5.** Photograph of 50 mL (a) DES; (b) DES with newly added LiCoO_2_ (c) the mixture after 30 mins; (d) the mixture after optimal leaching. (e) the supernatant after centrifugation.

**
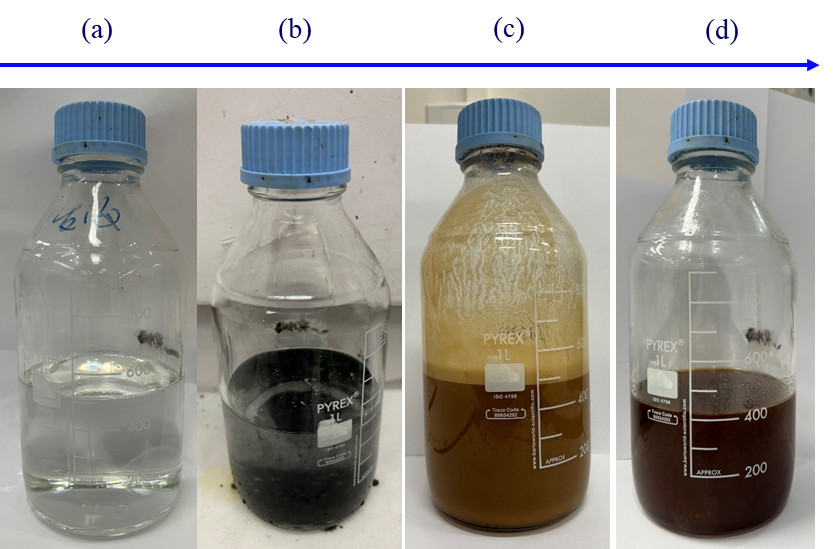
**

**Figure S6.** Photograph of 500 mL (a) DES; (b) DES with newly added LiCoO_2_. (c) the mixture after optimal leaching. (d) the supernatant after centrifugation.


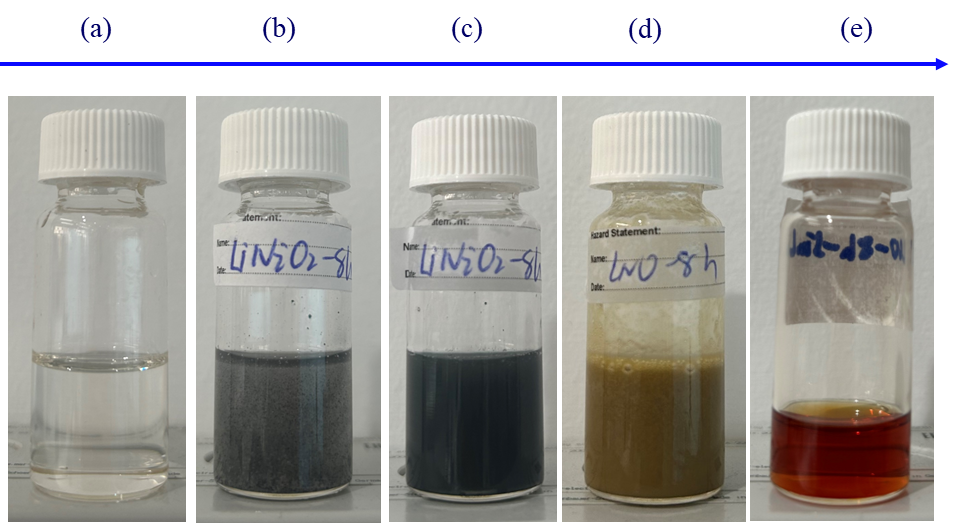


**Figure S7.** Photograph of 5 mL (a) DES; (b) DES with newly added LiNiO_2_. (c) the mixture after 1 h; (d) the mixture after optimal leaching. (e) the supernatant after centrifugation and filtration.

**
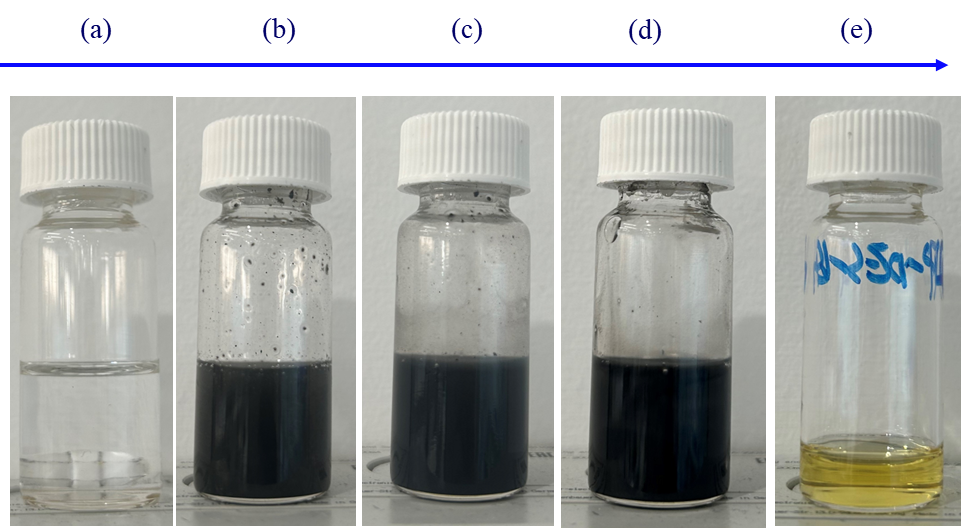
**

**Figure S8.** Photograph of 5 mL (a) DES; (b) DES with newly added LiFePO_4_. (c) the mixture after 1 h; (d) the mixture after optimal leaching. (e) the supernatant after centrifugation and filtration.


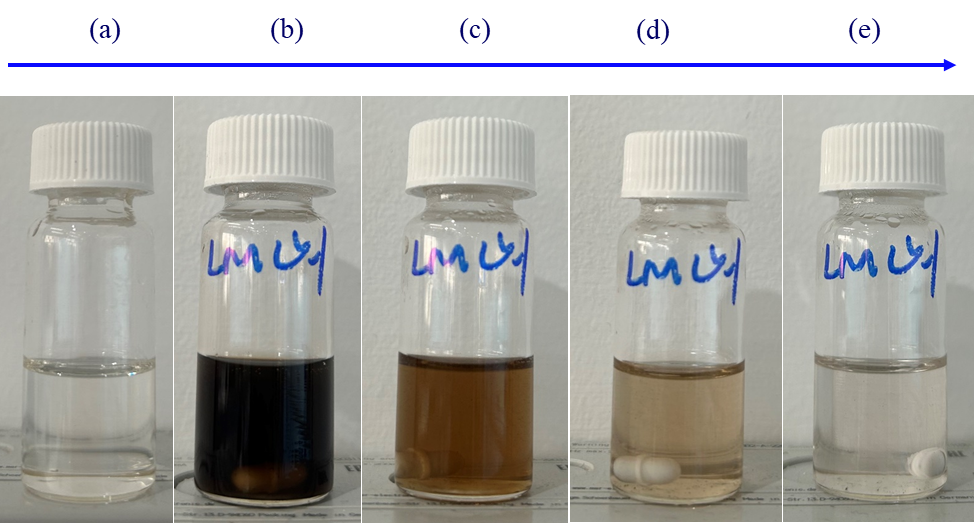


**Figure S9.** Photographs of 5 mL (a) DES; (b) DES with newly added LiMn_2_O_4_. (c) the mixture after 20 m; (d) the mixture after 30 m. (e) the mixture after 1 h.

**
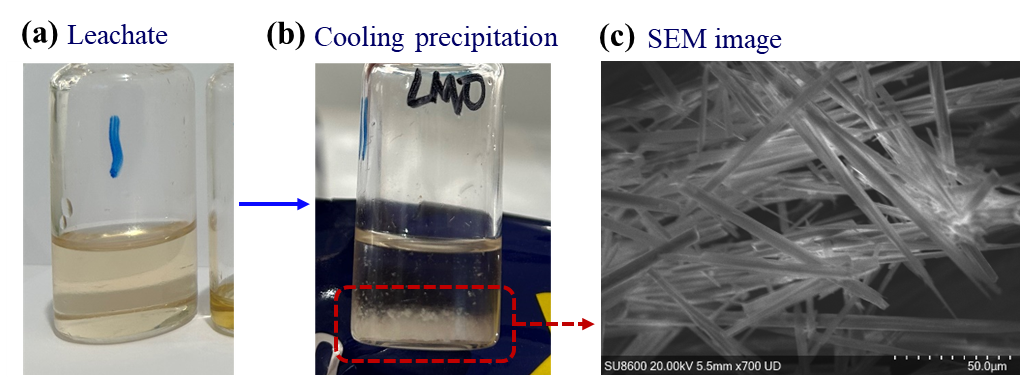
**

**Figure S10.** (a) Photograph of leachate after complete leaching of LiMnO_2_. (b) Photograph of the leachate with precipitate formed after cooling precipitation. (c) SEM image of the precipitate.

**
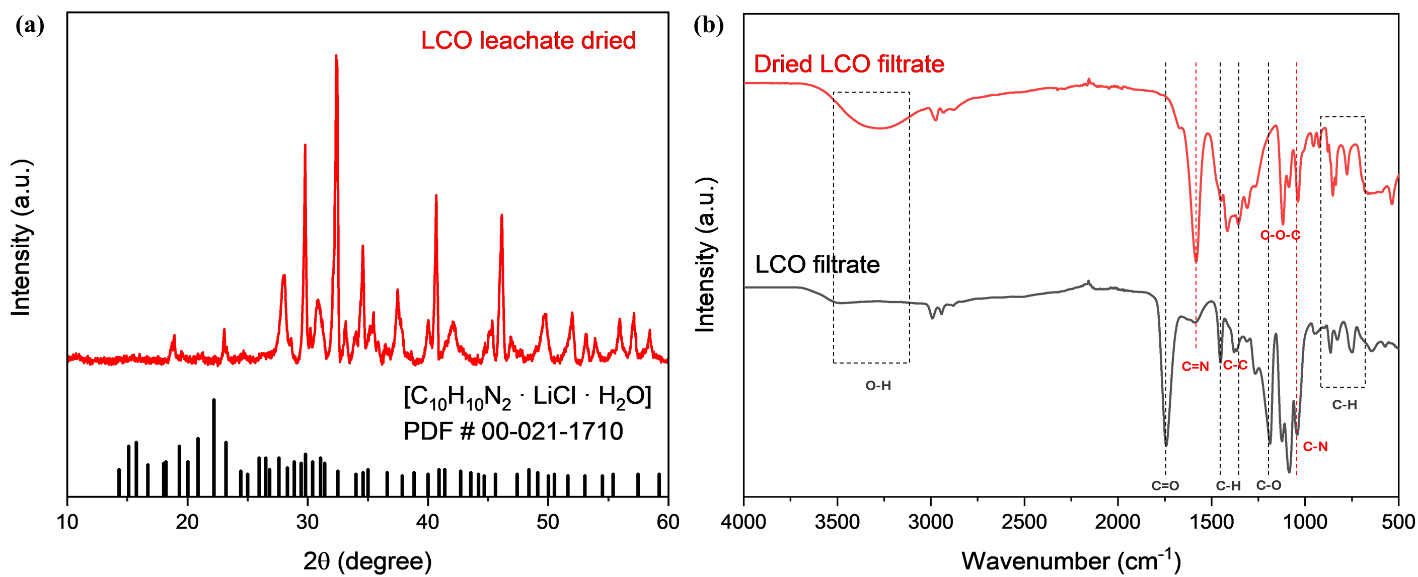
**

**Figure S11.** (a) XRD analysis of dried LCO filtrate. (b) FTIR spectra of LCO filtrate before and after drying.


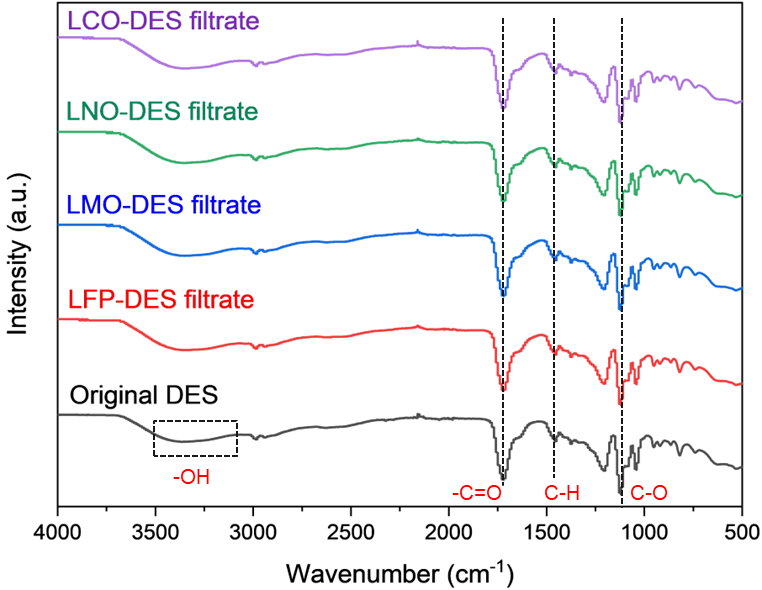


**Figure S12.** FTIR spectra of the original DES and DES leachates from commercial cathodes.

**
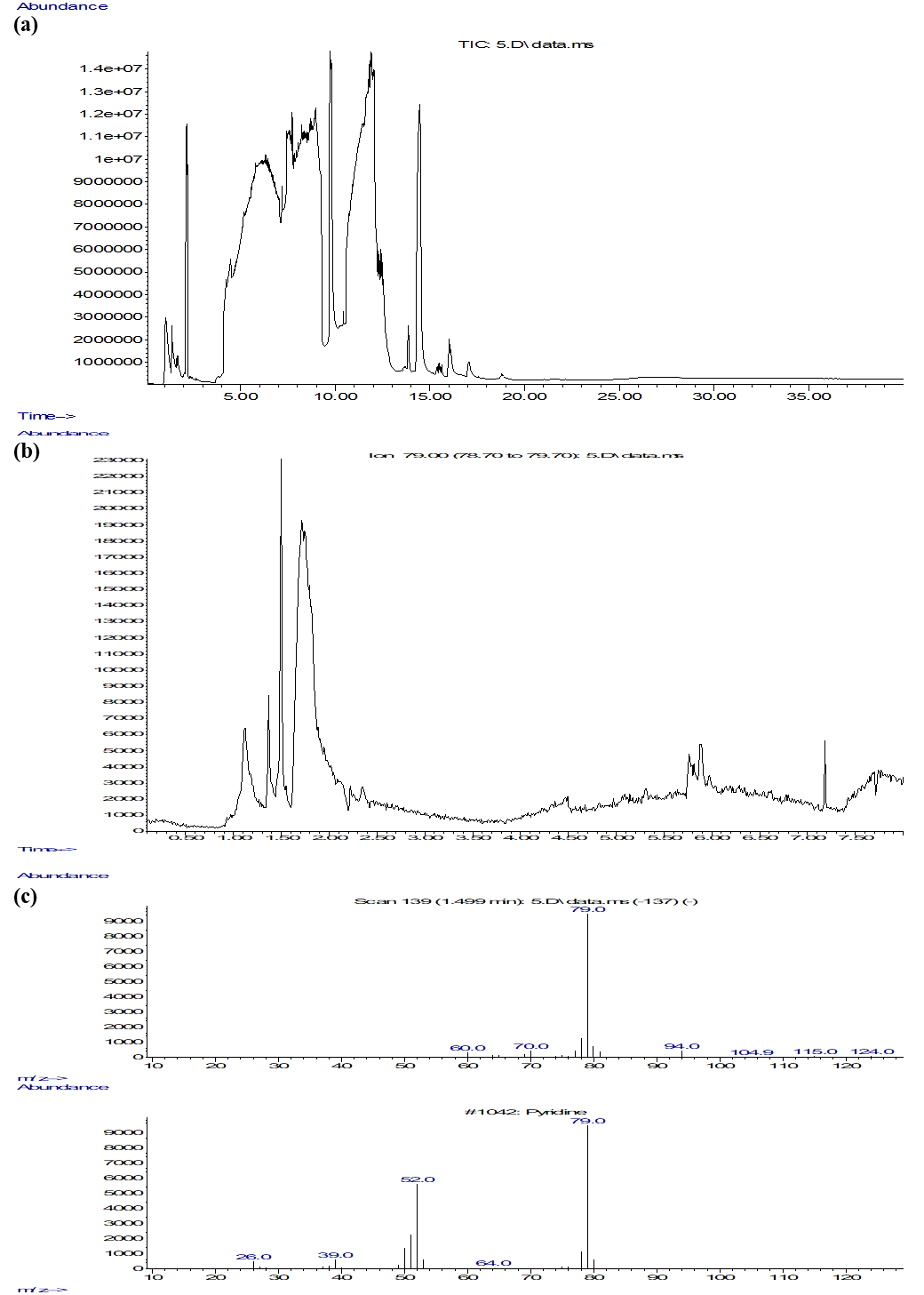
**

**Figure S13**. GC-MS analysis of a DES leaching filtrate. (a) Total ion chromatogram. (b) Ion chromatogram m/z 79 showing pyridine at 1.499 mins. (c) NIST Mass Spectral Library database comparison identifying pyridine.

**
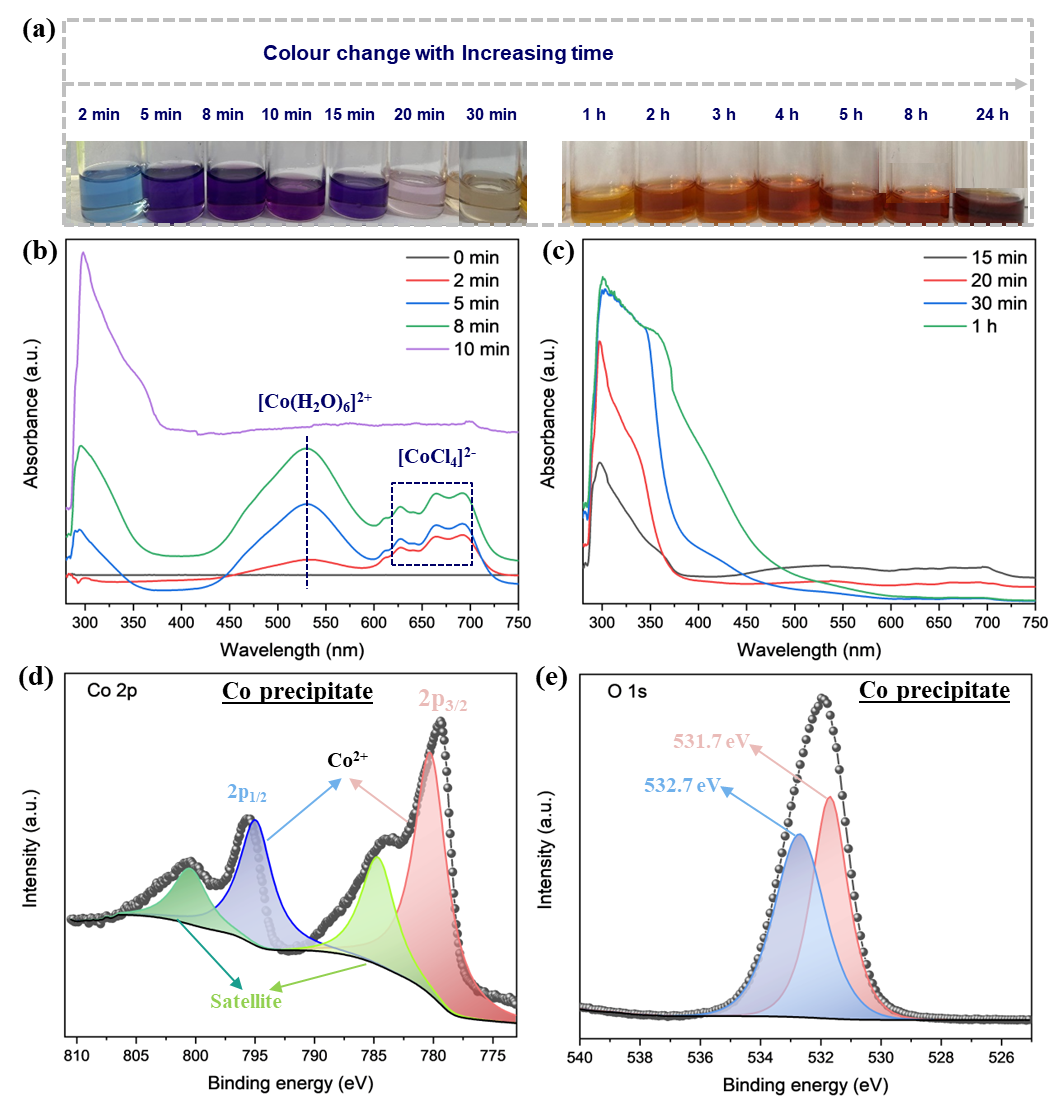
**

**Figure S14**. LiCoO_2_ dissolution in DES with cobalt precipitate.

(a) Colour change of the LCO leaching filtrates with time.

(b)-(c) UV/Vis spectra of filtrate: (b) within 10 mins, (c) 15 min to 1 h.

(d)-(e) XPS spectra of leached Co precipitate: (d) Co 2p, (e) O 1s.


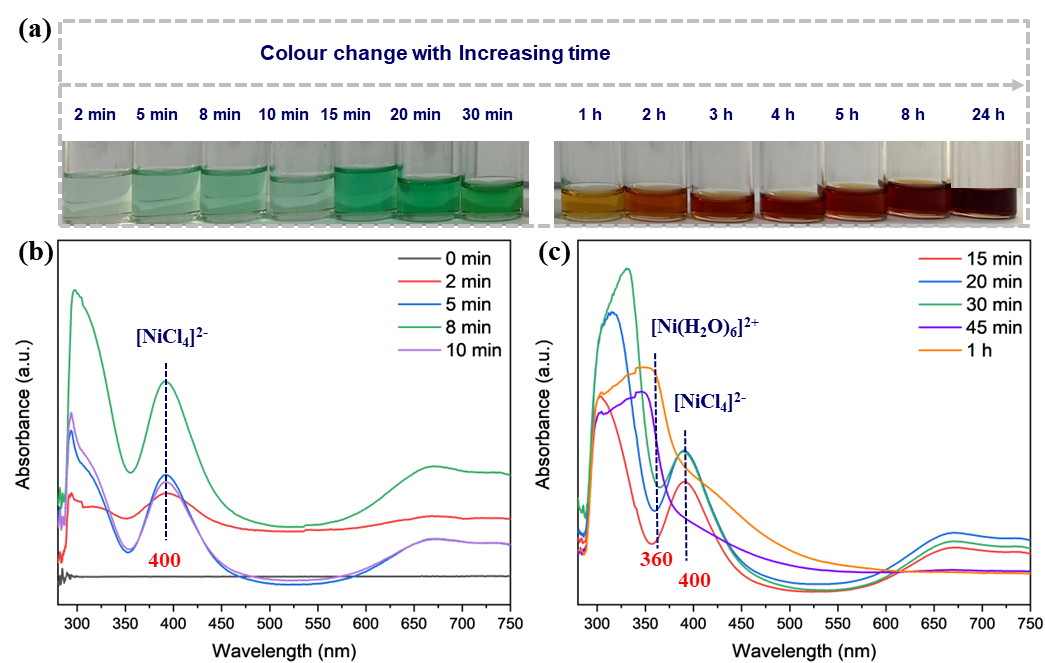


**Figure S15**. LiNiO_2_ dissolution in DES with nickel precipitate.

(a) Colour change of the LNO leaching filtrates with time.

(b)-(c) UV/Vis spectra of filtrate: (a) within 10 mins, (b) 15 min to 1 h.


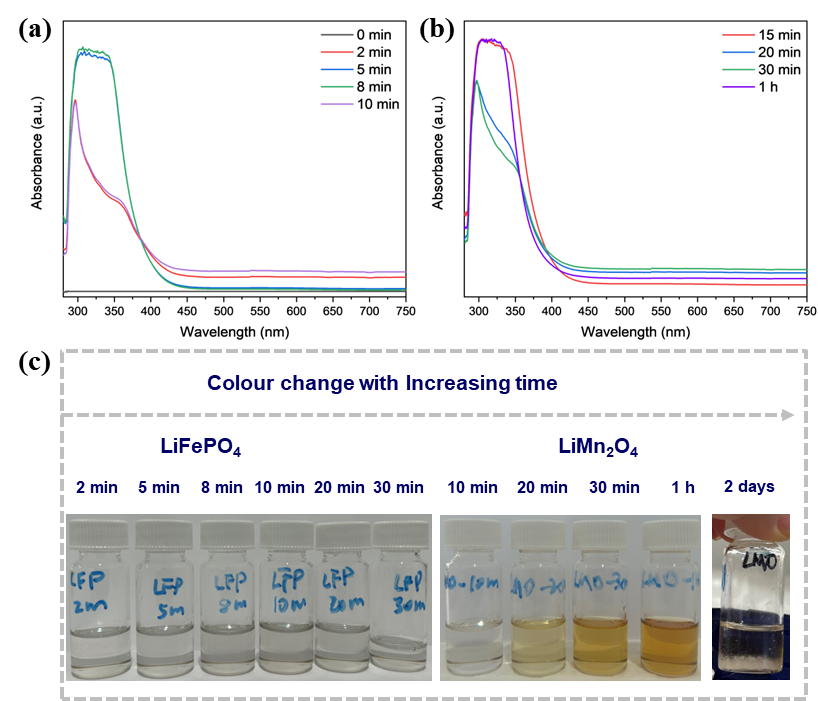


**Figure S16**. LiFePO_4_ and LiMn_2_O_4_ dissolution in DES.

(a)-(b) UV/Vis spectra of LFP filtrate: (a) within 10 mins, (b) 15 min to 1 h.

(a) Colour change of the LFP and LMO leaching filtrates with time.

**
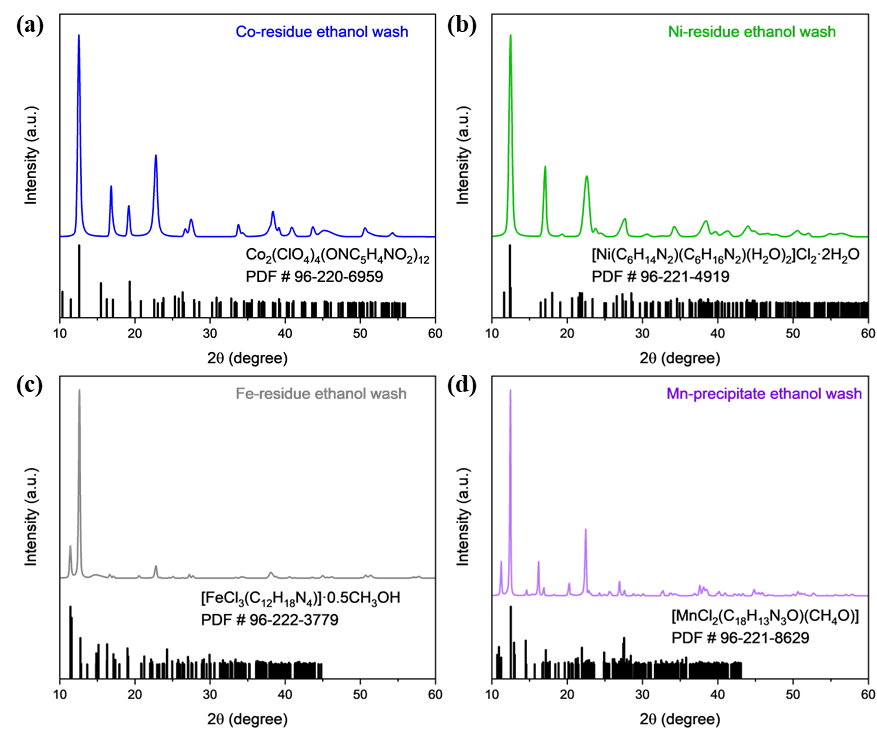
**

**Figure S17.** XRD analysis of the obtained precipitates from leaching of (a) LCO, (b) LNO, (c) LFP and (d) LMO.


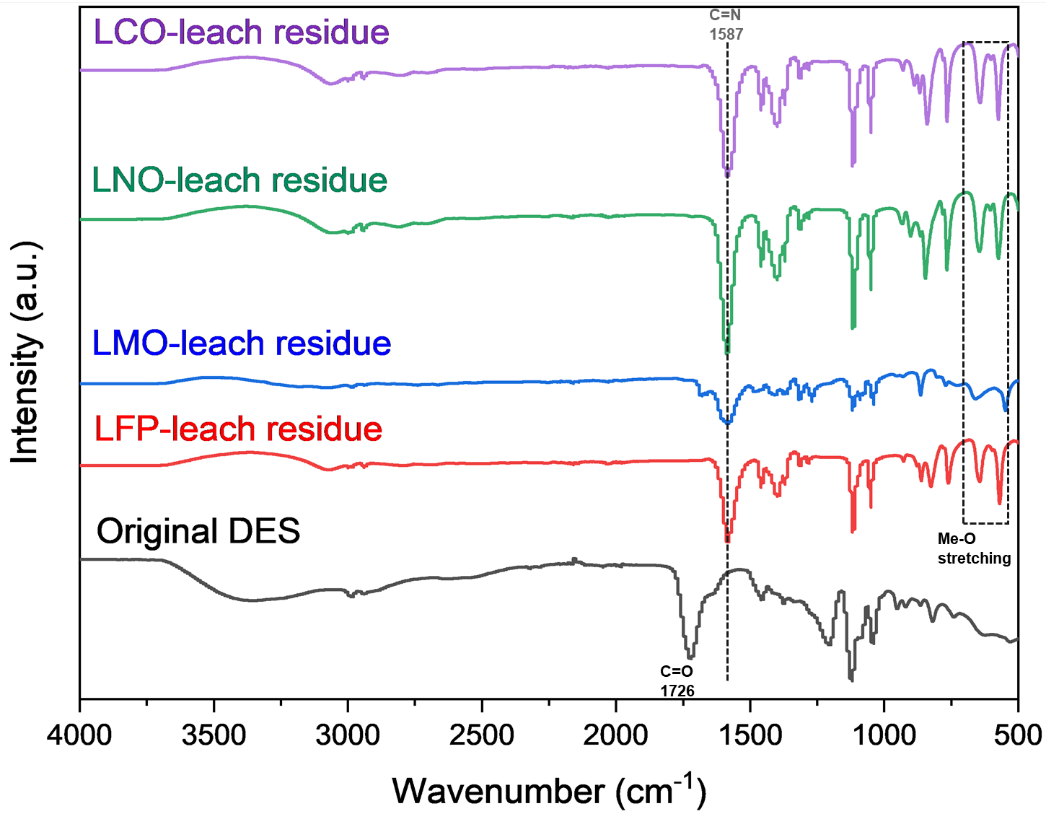


**Figure S18.** FTIR spectra of the leached residues from commercial cathodes.


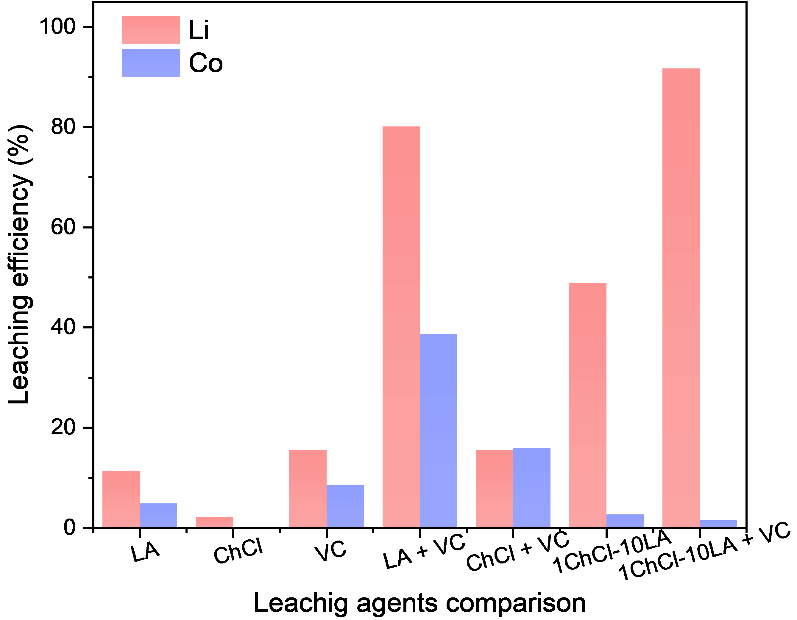


**Figure S19.** Comparison of the leaching efficiency of ChCl, LA, VC, ChCl+VC, LA+VC, 1ChCl-10LA, and 1ChCl-10LA+VC.

**
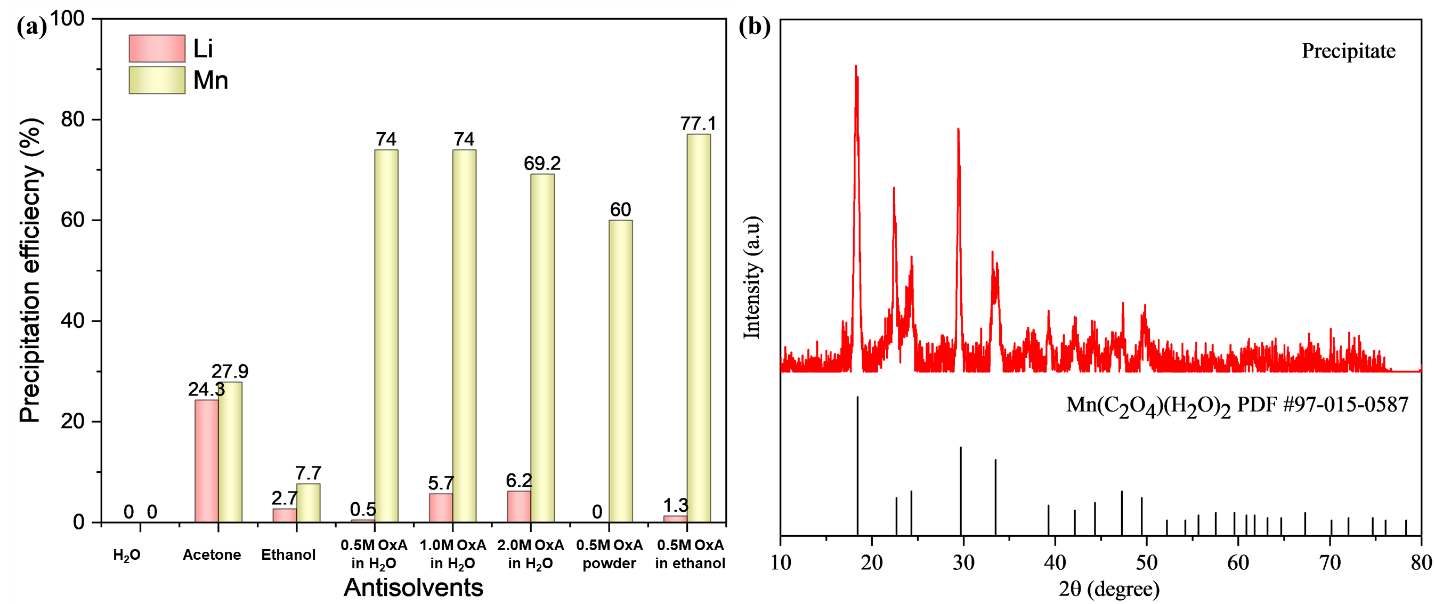
**

**Figure S20.** (a) Preliminary metal precipitation efficiency of a leachate by comparing several antisolvents. (b) XRD pattern of the precipitate obtained from antisolvent crystallization by 0.5 M oxalic acid in ethanol.


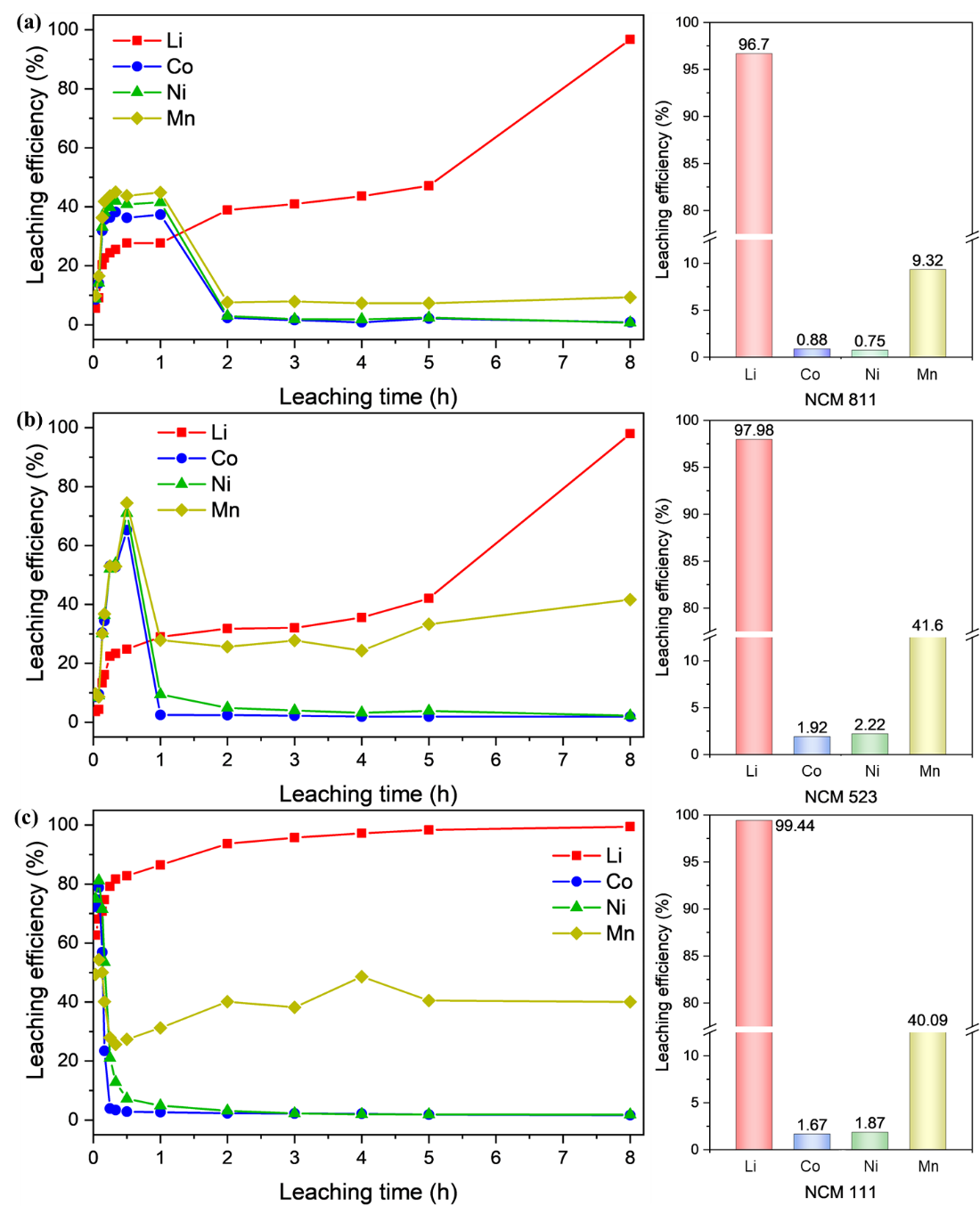


**Figure S21**. Variations of metal leaching efficiency with time and the leaching efficiency after 8 hours. (a) NCM811; (b) NCM523; (c) NCM111.


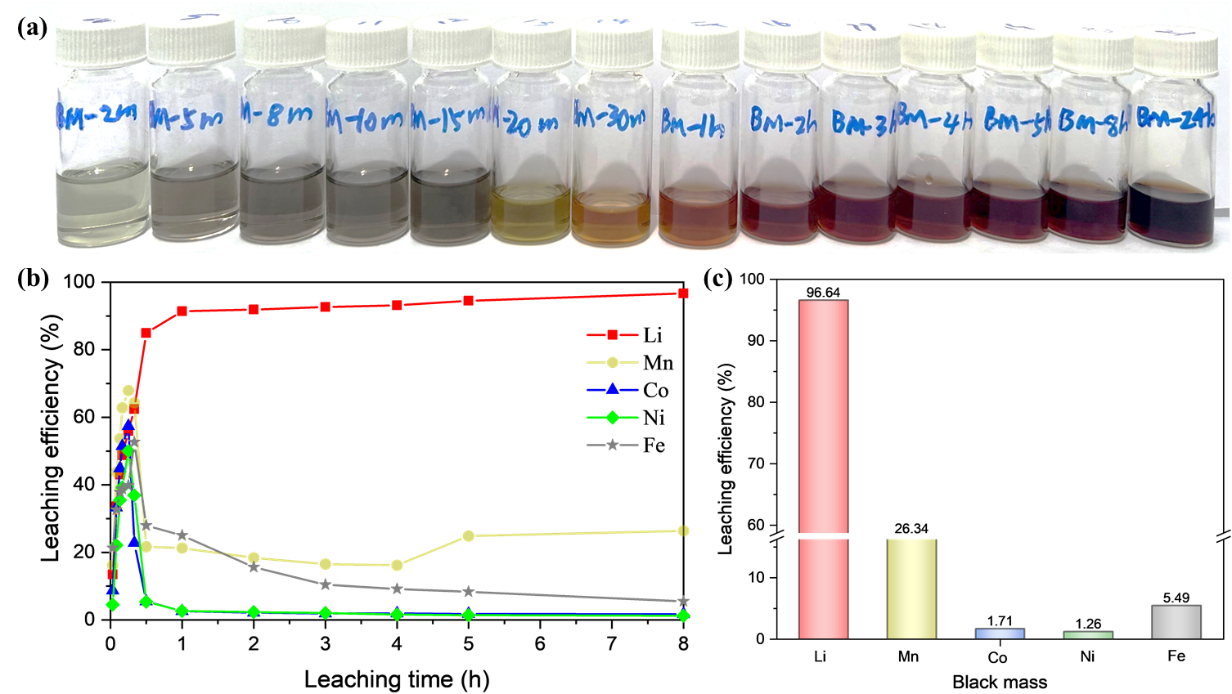


**Figure S22.** Black mass leaching process. (a) solution color changes, (b) metal leaching efficiency with time and (c) the leaching efficiency after 8 hours.

**
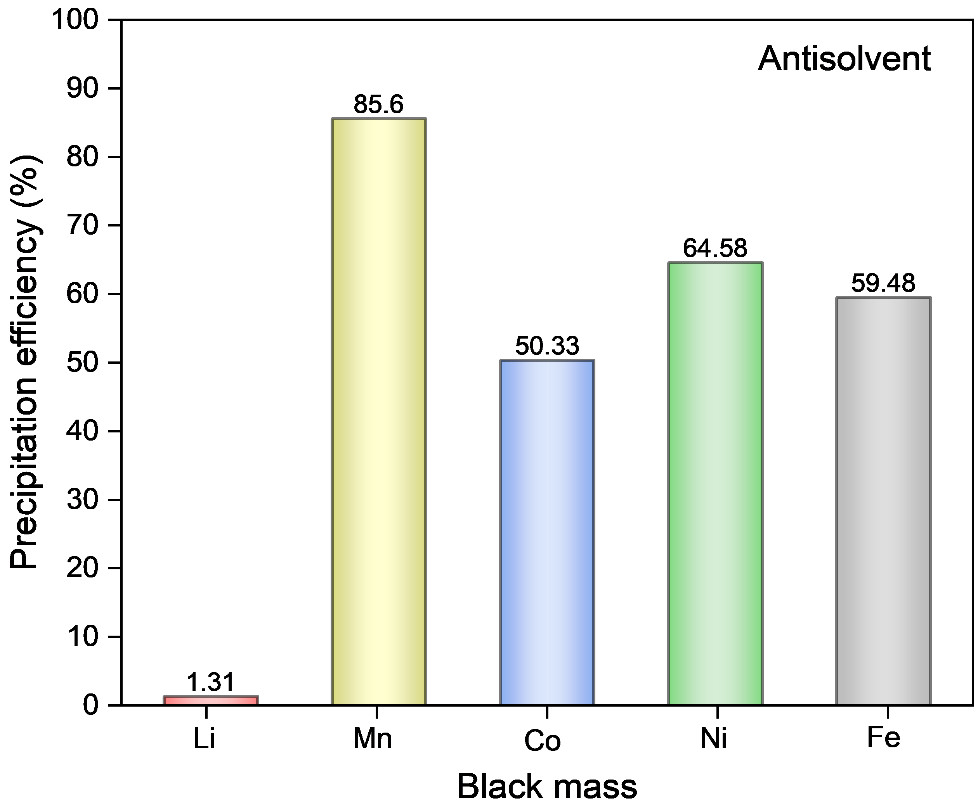
**

**Figure S23.** The precipitation efficiency of metals from black mass leachate after antisolvent crystallization.


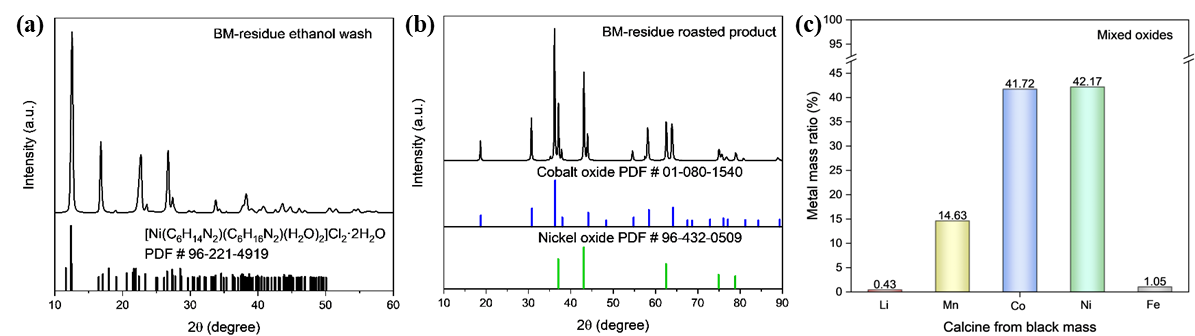


**Figure S24**. (a) XRD pattern of BM leaching residue, (b) XRD pattern of the calcined product, and (c) Metal mass ratio of the calcined product.


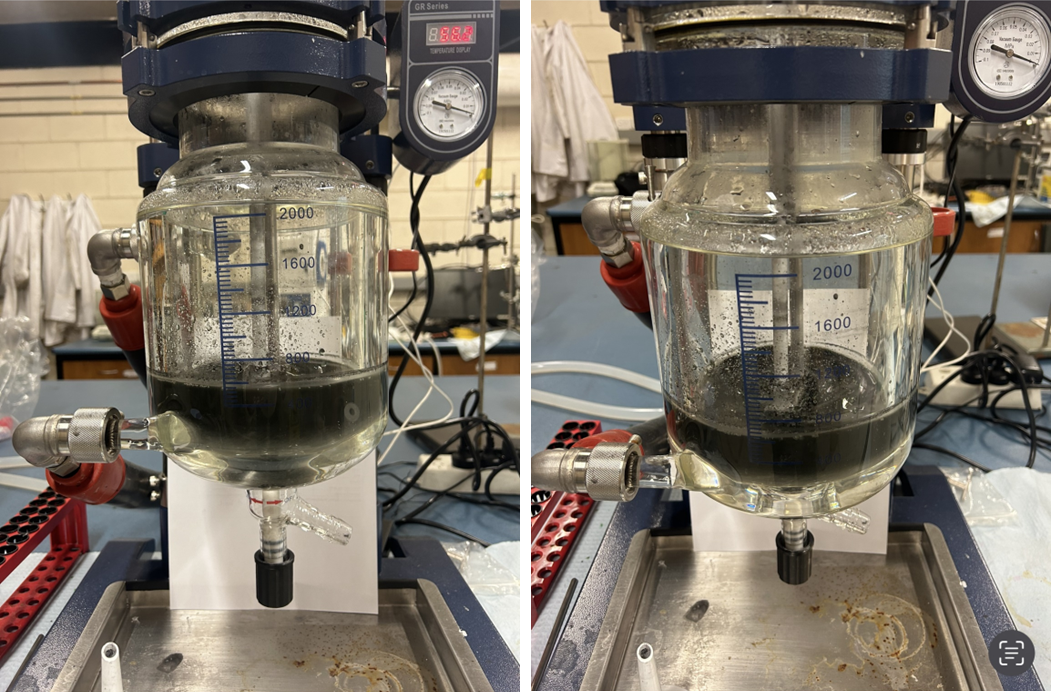


**Figure S25.** Scale-up experiments for leaching actual black mass.


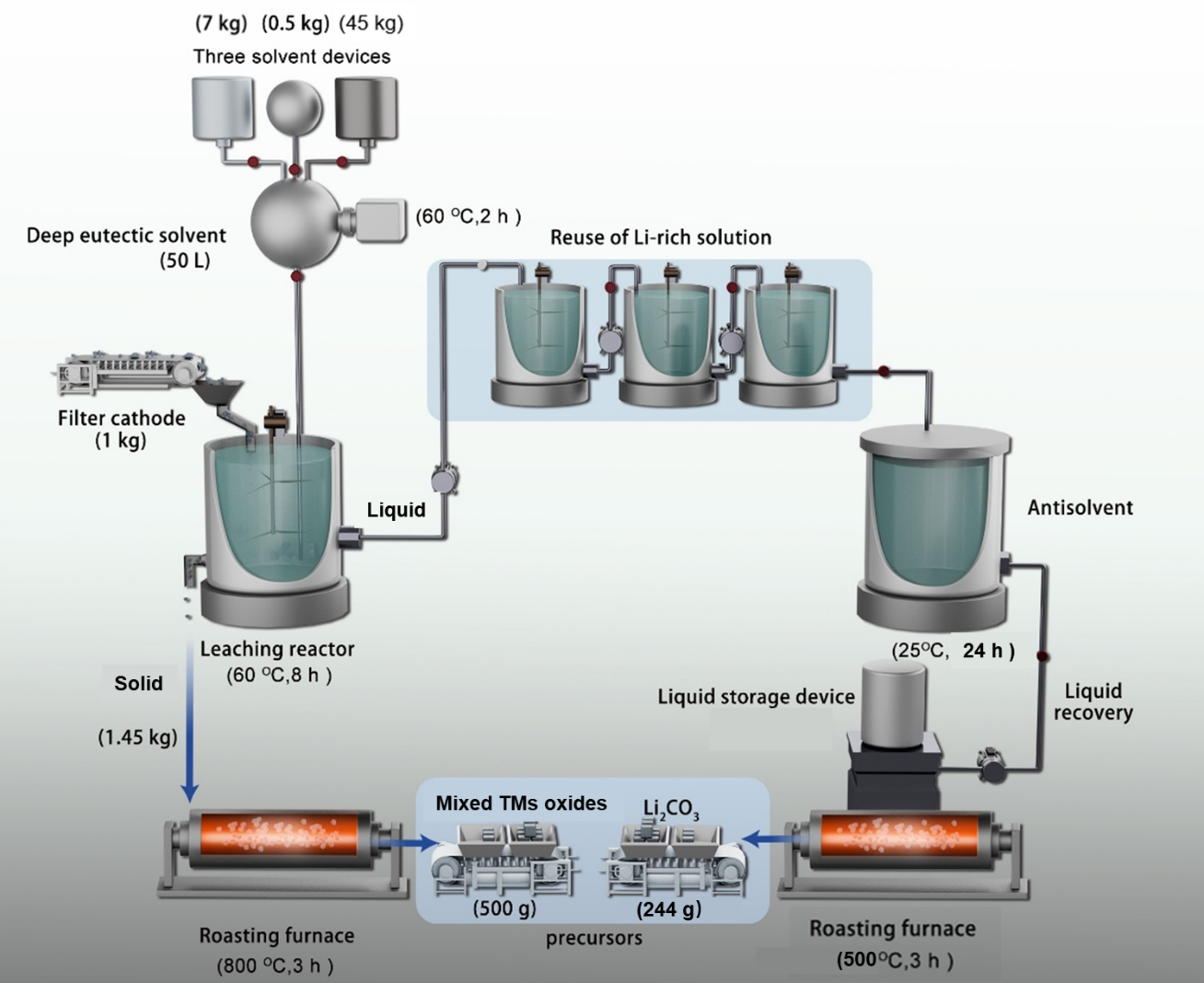


**Figure S26**. Proposed industrialization recycling schematic pathways for selective Li separation from spent LIBs cathodes via DES starting from DES preparation, leaching, DES circulation, antisolvent crystallization, and metal recovery.

**Table S1.** Metal contents of different spent LIBs cathode materials.

| **Cathodes** | **wt%** | | | | |
| --- | --- | --- | --- | --- | --- |
|  | **Li** | **Mn** | **Co** | **Ni** | **Fe** |
| LCO | 6.55 | - | 59.62 | - | - |
| LNO | 7.21 | - | - | 50.96 | - |
| LFP | 4.19 | - | - | - | 33.06 |
| LMO | 4.76 | 64.71 | - | - | - |
| NCM111 | 6.18 | 15.52 | 17.79 | 17.15 | - |
| NCM523 | 6.46 | 16.30 | 11.99 | 29.41 | - |
| NCM811 | 5.80 | 5.25 | 3.99 | 46.67 | - |
| Black mass | 4.87 | 7.57 | 14.34 | 17.69 | 1.62 |

**Table S2.** Metal leaching efficiency from various spent LIBs by similar DESs.

| **Cathode type** | **DES type** | **Conditions** | **Leaching efficiency %** | | | | **Ref.** |
| --- | --- | --- | --- | --- | --- | --- | --- |
|  |  |  | **Li** | **Co** | **Ni** | **Mn** |  |
| LiMn_2_O_4_ | 2ChCl-1LA | 105°C, 24 h | 100 | 100 | 100 |  | ^9^ |
| LiNi_0.5_Mn_1.5_O_4_ |  |  | ~100 | - | ~100 | ~100 |  |
| LiNi_0.8_Co_0.2_O_2_ |  |  | >85 | >85 | >85 | - |  |
| LCO | 1GUC-2LA-1wt%VC | 50 °C, 400 rpm, 24 h, S/L ratio of 1:50. | 97.4 | 96.9 | - | - | ^10^ |
| NCM111 |  |  | 100 | 97 | 95 | 96 |  |
| NCM523 |  |  | 100 | 89 | 87 | 85 |  |
| NCM622 |  |  | 100 | 95 | 93 | 93 |  |
| NCM811 |  |  | 100 | 93 | 100 | 100 |  |
| LCO | 1GUC-2LA | 80°C, 24 h, S/L 19.9 mg/g | 100 | 100 | - | - | ^11^ |
| LCO | 1BeCl-5LA | 120°C, 2.2 h, S/L 20 mg/g | 99.98 | 99.86 | - | - | ^12^ |
| LCO | 1ChCl-2EG | 180°C, 24 h, S/L 20 mg/g | >90 | >90 | - | - | ^13^ |
| LCO | 1ChCl-2Urea | 180°C, 12 h, S/L 20 mg/g | 94.7 | 97.9 | - | - | ^14^ |
| LCO | 1ChCl-2CA (35wt% H_2_O and Al, Cu) | 40°C, 1 h, S/L 20 mg/g | 93 | 98 | - | - | ^15^ |
| LCO | 1ChCl-1OA | 180 °C, 10 s, S/L 20 mg/3 g | 100 | 100 | - | - | ^16^ |
| LCO | 1ChCl-1OA | 90 °C, 3 h, S/L 300 mg/5 g | ~95 | ~98 | - | - | ^17^ |
| LCO | 1ChCl-2FA | 90 °C, 12 h, S/L 20 g/L | 99 | 99 | - | - | ^18^ |
| NCM111 | 1ChCl-2AA | 120 °C, 12 h, S/L 20 g/L | 100 | 100.5 | 101.3 | 99.2 | ^8^ |
| NCM811 | 1BeCl-9FA | 140 °C, 6 h, S/L 20 g/L | 98.0 | 94.2 | 92.4 | 96.0 | ^19^ |
| NCM111 | 2ChCl-1VC-6H_2_O | 50 °C, 1 h, S/L 0.1 g/2.5 g | 96.2 | 98.1 | 98.9 | 99.3 | ^20^ |
| LCO | ChCl–OA–8H_2_O | Microwave 100°C, 10 min, S/L 20 mg/mL | 99.1 | 0.8 | - | - | ^21^ |
| LCO | 1DMT-1OAD+30%H_2_O | 110°C, 2.5h, S/L 20 mg/mL | 100 | 0.14 | - | - | ^22^ |
| LCO | 5EG–1OAD | 90°C, 12 h, S/L 16 mg/mL | 94.4 | <1.2 | - | - | ^23^ |
| Li_14.8_Ni_1.7_Co_8.5_MnO_30.5_ |  |  | 94.1 | <1.2 | <1.2 | <1.2 |  |
| LCO | 5EG–1TA | Hydrothermal 120°C, 12 h, S/L 20 mg/mL | 98.34 | 2.0 | - | - | ^24^ |
| Li_3.2_Ni_2.4_Co_1.0_Mn_1.4_O_8.3_ |  |  | 98.86 | 1.92 | 3.62 | 3.03 |  |
| LCO | 1ChCl-10LA-VC | 60°C, 8 h, S/L 20 mg/mL | 98.2 | 1.4 | - | - | This work |
| NCM811 |  |  | 96.70 | 0.88 | 0.75 | 9.32 |  |
| Black mass |  |  | 96.64 | 1.71 | 1.26 | 26.3 |  |

**Note:** Guanidine hydrochloride=GUC; BeCl=Betaine hydrochloride; Ethylene glycol=EG; CA=Citric acid; dimethylthetin=DMT; Oxalic acid dihydrate=OAD; Acetic acid=AA; Tartaric acid=TA.

**Table S3.** Price of some DESs calculated in processing 1 kg of cathode materials (chemicals price from Alibaba).

| **DES type** | **Cost (USD/kg)** | **DES reuse** | **Ref.** |
| --- | --- | --- | --- |
| 2ChCl−1LA | 53.2 | - | ^9^ |
| GUC-LA (1:2)-1wt%VC | 64.45 | 3 | ^10^ |
| 1GUC-2LA | 63.2 | 2 | ^11^ |
| 1BeCl−5LA | 69.4 | - | ^12^ |
| 1ChCl-2EG | 50 | 2 | ^13^ |
| 1ChCl-2Urea | 35.2 | - | ^14^ |
| 1ChCl-2CA (35wt% H_2_O and Al, Cu) | 32.7 | - | ^15^ |
| 1ChCl-1OA | 123 | 3 | ^16^ |
| 1ChCl-1OA | 13.9 | 3 | ^17^ |
| 1ChCl-2FA | 58.7 | - | ^18^ |
| 1ChCl-2AA | 39.2 | 3 | ^8^ |
| 1BeCl-9FA | 76.1 | - | ^19^ |
| 2ChCl-1VC-6H_2_O | 32.6 | - | ^20^ |
| ChCl–OA–8H_2_O | 27.1 | 5 | ^21^ |
| 1DMT-1OAD+30%H_2_O | 236.2 | 1 | ^22^ |
| 5EG–1OAD | 44.5 | 3 | ^23^ |
| 5EG–1TA | 67.9 | 5 | ^24^ |
| 1ChCl-10LA-VC | 62.25 | 4 | This work |

**References**

1 P. E. Blöchl, *Phys. Rev. B*, 1994, **50**, 17953–17979.

2 G. Kresse and D. Joubert, *Phys. Rev. B - Condens. Matter Mater. Phys.*, 1999, **59**, 1758–1775.

3 G. Kresse and J. Furthmüller, *Comput. Mater. Sci.*, 1996, **6**, 15–50.

4 G. Kresse and J. Furthmüller, *J. Phys. Chem. A*, 1996, **54**, 11169–11186.

5 J. P. Perdew, K. Burke and M. Ernzerhof, *Phys. Rev. Lett.*, 1996, **77**, 3865–3868.

6 S. Grimme, J. Antony, S. Ehrlich and H. Krieg, *J. Chem. Phys.*, 2010, **132**, 154104.

7 R. Hosseinzadeh, S. Zarei, Z. Valipour and B. Maleki, *Heliyon*, 2024, **10**, e37170.

8 Y. Lyu, J. A. Yuwono, Y. Fan, J. Li, J. Wang, R. Zeng, K. Davey, J. Mao, C. Zhang and Z. Guo, *Adv. Mater.*, 2024, 1689–1699.

9 R. Morina, D. Callegari, D. Merli, G. Alberti, P. Mustarelli and E. Quartarone, *ChemSusChem*, 2022, **15**, e202102080.

10 Q. Yan, A. Ding, M. Li, C. Liu and C. Xiao, *Energy and Fuels*, 2023, **37**, 1216–1224.

11 Y. Tian, W. Chen, B. Zhang, Y. Chen, R. Shi, S. Liu, Z. Zhang and T. Mu, *ChemSusChem*, 2022, **15**, 1–8.

12 Y. Luo, C. Yin and L. Ou, *ACS Sustain. Chem. Eng.*, 2023, **11**, 11834–11842.

13 M. K. Tran, M. T. F. Rodrigues, K. Kato, G. Babu and P. M. Ajayan, *Nat. Energy*, 2019, **4**, 339–345.

14 S. Wang, Z. Zhang, Z. Lu and Z. Xu, *Green Chem.*, 2020, **22**, 4473–4482.

15 N. Peeters, K. Binnemans and S. Riaño, *Green Chem.*, 2020, **22**, 4210–4221.

16 T. Li, Y. Xiong, X. Yan, T. Hu, S. Jing, Z. Wang and X. Ge, *J. Energy Chem.*, 2022, **72**, 532–538.

17 T. Hu, T. Li, X. Liu, Z. Wang, L. Lou, S. Jing, X. Yan, Y. Xiong, J. Xiong and X. Ge, *Green Chem.*, 2024, **26**, 2653–2660.

18 L. Chen, Y. Chao, X. Li, G. Zhou, Q. Lu, M. Hua, H. Li, X. Ni, P. Wu and W. Zhu, *Green Chem.*, 2021, **23**, 2177–2184.

19 C. Liu, J. Yu, J. Hu, J. Xu, A. Yu, T. Liu, Z. Wang, X. Luo, C. Deng, F. Luo, J. He and G. Zeng, *J. Environ. Chem. Eng.*, 2024, **12**, 17–20.

20 Y. Hua, Y. Sun, F. Yan, S. Wang, Z. Xu, B. Zhao and Z. Zhang, *Chem. Eng. J.*, 2022, **436**, 133200.

21 W. Ma, M. Liu, X. Zhang, Q. Zhao and Z. Liang, *Energy and Fuels*, 2023, **37**, 724–734.

22 Y. Luo, C. Yin and L. Ou, *Sci. Total Environ.*, 2023, **902**, 166095.

23 S. Tang, M. Zhang and M. Guo, *ACS Sustain. Chem. Eng.*, 2022, **10**, 975–985.

24 Z. Yang, S. Tang, X. Huo, M. Guo and M. Zhang, *Environ. Res.*, 2023, **233**, 116337.
